# Wound Healing Activity of Iron Oxide Nanoparticles; Comparative In Vivo Study on *Staphylococcus aureus*-Infected and Non-Infected Wounds

**DOI:** 10.3390/antibiotics15060584

**Published:** 2026-06-08

**Authors:** Marwa Reda Bakkar, Alaa M. Ali, Gehad E. Elkhouly, Nermeen R. Raya, Kareem A. Abdelmeguid, Shimaa K. Mohamed, Yasmin Abo-zeid

**Affiliations:** 1 Botany and Microbiology Department, Faculty of Science, Capital University, Ain Helwan, Cairo 11795, Egypt; marwa_mahmoud01@science.capu.edu.eg (M.R.B.); kareem.abdelmeguid@lu.lv (K.A.A.); 2 Department of Pathology, Faculty of Veterinary Medicine, Cairo University, Giza 12211, Egypt; alaamohamedali13@cu.edu.eg; 3 Department of Pharmaceutics and Industrial Pharmacy, Faculty of Pharmacy, Capital University, Ain Helwan, Cairo 11795, Egypt; gehad.elkhouly@pharm.capu.edu.eg (G.E.E.); nermeen.rayh@pharm.capu.edu.eg (N.R.R.); 4 Faculty of Life Sciences and Medicine, University of Latvia, Jelgavas iela 1, LV-1004 Riga, Latvia; 5 Department of Pharmacology and Toxicology, Faculty of Pharmacy, Capital University, Ain Helwan, Cairo 11795, Egypt; shimaa_kamal@pharm.capu.edu.eg; 6 Department of Pharmaceutics, UCL School of Pharmacy, University College London, 29–39 Brunswick Square, London WC1N 1AX, UK

**Keywords:** iron oxide nanoparticles, multidrug-resistant bacteria, antibiotic resistance, infected wounds, wound healing, antibacterial activity

## Abstract

**Background/Objectives:** Wound infections represent a major category of healthcare-associated infections (HAIs) that interrupt the wound healing process, resulting in delayed wound healing and increasing the incidence of mortality, morbidity and healthcare costs. With the emergence of antibiotic resistance, there is an urgent need to find alternative therapeutic strategies capable of overcoming antibiotic resistance while simultaneously promoting wound healing. Previously, we synthesized iron oxide nanoparticles stabilized with cetyltrimethylammonium bromide (IONPs-CTAB), reported their antimicrobial activity against selected multidrug-resistant bacteria (MDR-bacteria) and SARS-CoV2 virus, and addressed their biocompatibility with the skin and eyes of rabbits. Therefore, it is hypothesized that IONPs-CTAB might be a promising alternative therapeutic agent for management of infected wounds. **Methods:** IONPs-CTAB were synthesized, and their successful synthesis was confirmed by FTIR, DSC-TGA, and XPS. Their antibacterial activity against three MDR-bacteria, *Staphylococcus aureus* (*S. aureus*), methicillin-resistant *Staphylococcus aureus* (MRSA) and *Escherichia coli* (*E. coli*), isolated from infected wounds was investigated via the microdilution test to determine MIC/MBC, and a time–kill curve study was also performed. Subsequently, an in vivo study was conducted to assess their wound healing activity on both non-infected and infected wounds. **Results:** IONPs-CTAB had MIC and MBC values ranging from 125 to 250, and 500 to 1000 µg/mL, respectively. The time–kill curve study showed an effective control of bacterial growth for all tested bacteria. The vivo study demonstrated the superior wound healing activity of IONPs-CTAB compared to standard treatment on both non-infected and infected wounds. This was further confirmed by histopathological examination and biochemical analysis. **Conclusions:** IONPs-CTAB might be a good therapeutic alternative for the management of infected and non-infected wounds. However, future studies are still required to assess their long-term safety and the possibility of their extravasation to systemic circulation, with their potential accumulation in various organs after a long-term application.

## 1. Introduction

Wound healing is a complicated biological process essential for restoring skin’s integrity to assure the body’s protective barrier against the external environment [1,2]. Based on healing time, wounds are classified into acute and chronic wounds. Acute wounds usually heal within a predictable and well-defined timeframe; however, several factors govern the rate of wound healing, such as wound severity, including its size and depth. This is contrary to chronic wounds, which are characterized by a delayed healing process [3]. Chronic wounds fail to heal in patients suffering from venous and arterial insufficiency, age, obesity, or diabetes, as well as in immunocompromised patients [4]. The delay of healing is linked to wound infections occurring in these patients; this is associated with significant pain and a marked reduction in quality of life, increasing the incidence of amputation, morbidity and mortality [5]. To develop an effective therapeutic agent that can control wound infection and promote wound healing, it is important to understand the different phases of wound healing and how infections affect these phases and interrupt the wound healing process.

The healing process involves several phases that might overlap; they are hemostasis, inflammation, proliferation, and remodeling [4,6,7]. Among these phases, the inflammatory phase is particularly crucial, as it massively affects both the rate and overall success of wound healing [4,6]. The incidence of injury is followed by platelet aggregation, which initiates the hemostasis phase; this involves the release of chemotactic mediators including transforming growth factor-β1 (TGF-β1) and platelet-derived growth factor (PDGF). These mediators recruit inflammatory cells (e.g., monocytes that differentiate to pro-inflammatory macrophages) to the wound site to prevent incidence of infection. Afterwards, the initiation of proliferation and remodeling phases occurs through both resident and infiltrating cells via secreting a variety of growth factors, cytokines and chemokines to promote the proliferation and migration of fibroblasts and keratinocytes, thus regulating tissue repair, re-epithelization, and extracellular remodeling, and promoting wound contraction [8]. The incidence of wound infections is accompanied by persistent inflammation that impedes progression to the proliferative and remodeling phase, resulting in a very delayed wound healing process [4].

Several strategies have been explored for the management of chronic wounds. One approach involves the development of exosome-laden, oxygen releasing cryogel [9], which has been reported to promote collagen deposition, re-epithelization, and neo-vascularization, and reduce oxidative stress in diabetic wounds. However, this platform faces significant challenges in large-scale production [10]. Other investigations have focused on composite hydrogels incorporating antioxidative agents, including (poly)dopamine-modified gelatin together with an antimicrobial carbon nanotube and doxycycline, for the management of infected chronic wounds [11]. Despite their potential, these systems require near-infrared (NIR) irradiation to activate the photothermal antimicrobial activity of carbon nanotubes, and the prolonged exposure to doxycycline may contribute to antimicrobial resistance. Thermos-response hydrogels containing silver nanoparticles have also been explored, but their antibacterial activity was limited to *S. aureus* and was inactive against *P. aeruginosa* [12]. With the increasing prevalence of multidrug-resistant bacteria (MDR-bacteria) and failure of current antibiotics to resolve wound infections, there is an urgent need to find safe and effective therapeutic alternatives [7,13,14,15,16].

Natural compounds have been widely investigated for their therapeutic potential in promoting wound healing [17,18,19,20], and several studies have also demonstrated their antimicrobial activity against MDR-bacteria [21]. In parallel, metal-based nanoparticles have gained considerable attention as alternative antimicrobial agents for controlling resistant pathogens [22] and as versatile therapeutic platforms for wound management [23,24,25]. For example, nanoparticles of copper oxide, silver, and zinc oxide had proven antimicrobial activity against resistant bacteria [26,27] and were also applied for the management of infected diabetic wounds [7]. However, the toxicity of metal-based nanoparticles was also reported, limiting their application clinically [22,24].

In contrast, iron oxide nanoparticles (IONPs) have been reported to exhibit broad-spectrum antimicrobial activity against MDR-bacteria, with minimal or no potential for developing microbial resistance [22,28,29]. Their compatibility with biological systems is well established, as certain IONP formulations have been approved by the Food and Drug Administration (FDA) for the treatment of anemia [30]. Although the potential application of IONPs in wound healing management has been suggested in the literature [31,32,33], only a limited number of studies have evaluated their wound healing effects, and these investigations have primarily examined IONPs in combination with other biological materials rather than as standalone therapeutic agents [34,35,36,37].

According to authors’ knowledge, the in vivo wound healing activity of IONPs alone has not been previously investigated in the literature. The current study is the first to evaluate the wound healing efficacy of IONPs in both non-infected and *S. aureus*-infected wounds. Previously, we synthesized IONPs stabilized with cetyltrimethylammonium bromide (IONPs-CTAB) and reported their antimicrobial activity against MDR-bacteria; *S. aureus* and *E. coli*, and SARS-CoV-2 virus. This antimicrobial effect was attributed to the nanoparticles’ ability to generate reactive oxygen species (ROS), suggesting a reduced likelihood of inducing microbial resistance. We also established the dermal and ocular compatibility of IONPs-CTAB in a rabbit model, confirming their safety on intact and abraded skin as well as in ocular tissues [38]. Collectively, these findings indicate that IONPs-CTAB represent a promising therapeutic candidate for the management of infected wounds.

Based on this, a new batch of IONPs-CTAB were produced, and their successful synthesis was confirmed via FTIR, DSC-TGA, and XPS analysis. In addition, their morphological examination was conducted using Transmission Electron Microscopy (TEM), and their physicochemical properties including particle size, PDI, and zeta potential were also assessed with a Malvern Instrument. The antibacterial activity of IONPs-CTAB was assessed against MDR-resistant bacterial wound infections, namely *Staphylococcus aureus (S. aureus)*, methicillin-resistant *Staphylococcus aureus* (MRSA) and *Escherichia coli* (*E. coli*), which were collected from a clinical setting in Cairo, Egypt. This involved a microdilution method to determine the minimum inhibitory concentration (MIC) and minimum bactericidal concentration (MBC), followed by conducting a time–kill curve study. Subsequently, the wound healing potential of IONPs-CTAB at their compatible concentration (125 µg/mL) with the skin and eyes of rabbits was assessed in vivo on both non-infected, and *S. aureus*-infected wounds. Histopathological and biochemical examinations were also conducted to ascertain their wound healing efficacy. The results obtained demonstrate that IONPs-CTAB effectively controlled the growth of MDR-bacteria and promoted wound healing in both non-infected and *S. aureus*-infected wounds. However, further studies evaluating their long-term safety application and monitoring the potential of their accumulation in the systemic circulation and various organs are still essential for their clinical translation.

## 2. Results and Discussion

### 2.1. Synthesis and Characterization of IONPs-CTAB

**Figure 1:**
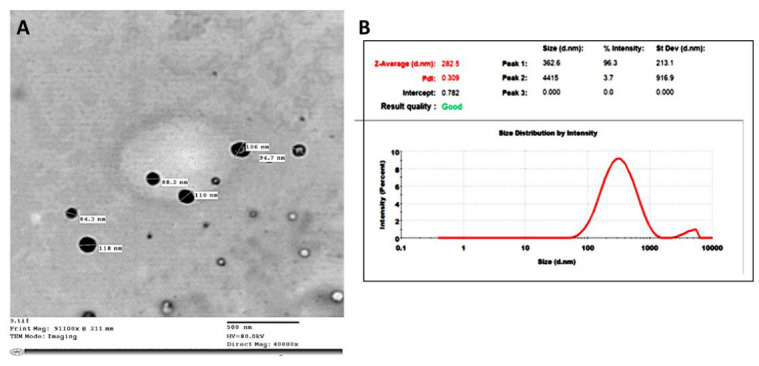
(**A**) Transmission Electron Microscopy (TEM) image of IONPs-CTAB; scale bar 100 nm; (**B**) dynamic light scattering (DLS) particle size and polydispersity index (PDI) of IONPs-CTAB.

The newly synthesized batch of IONPs-CTAB was verified through FTIR, DSC-TGA, and XPS analysis (Figures S1–S3 in Supplementary Materials), demonstrating a full agreement with our previously reported characterization [38]. DLS measurements indicated a mean hydrodynamic diameter of 282.5 ± 11.22 nm, a PDI of 0.309, and a zeta potential of 9.12 ± 1.20 mV, consistent with earlier findings [38]. Although the zeta potential exhibited relatively low negativity, the dispersion remained stable, as indicated by PDI ≤ 0.3, reflecting a mono-disperse system with minimal aggregation [39,40,41]. This reduced zeta potential is attributed to the adsorption of the non-toxic cationic surfactant CTAB on the surface of nanoparticles to stabilize IONPs [38]. TEM imaging (Figure 1) confirmed the spherical morphology of nanoparticles and showed no evidence of aggregation. As expected, the particle size observed using TEM was smaller than the hydrodynamic diameter measured with DLS, a well-documented outcome arising from differences in measurement principles and sample preparation techniques [38,41,42,43,44,45].

### 2.2. Microbiological Studies

#### 2.2.1. Antibacterial Activity

The antibacterial activity of IONPs-CTAB colloidal dispersion was determined quantitatively by estimating values of MIC and MBC, and results are presented in Table 1. As revealed, Gram-positive isolates, *S. aureus* and MRSA, were more susceptible to IONPs-CTAB than Gram-negative bacteria. MIC and MBC values recorded for *S. aureus* and MRSA were similar and were equivalent to 125 µg/mL and 500 µg/mL, respectively. This contrasts with the higher MIC and MBC values recorded for *E. coli*: 250 µg/mL and 1000 µg/mL, respectively.

The obtained data are consistent with our previous publication [38], where IONPs-CTAB demonstrated a higher antibacterial activity against *S. aureus* than *E. coli.* The antibacterial activity of IONPs-CTAB was linked to their potential to release reactive oxygen species (ROS). The low sensitivity observed with Gram-negative bacteria may be linked to the cell wall structure, as Gram-negative bacteria have an additional lipid outer membrane bilayer that is resistant to antimicrobial agents due to the presence of a lipopolysaccharide layer [26,42]. Moreover, the additional lipid outer membrane bilayer was reported to act as a barrier, reducing the permeation of ROS, and this could render them less sensitive to IONPs-CTAB (Bhuiyan et al. 2020 [46]; Ali et al. 2025b [38]; Hanada et al. 2026 [47]).

The MBC/MIC ratio was calculated for each tested bacterium to assess the bactericidal/bacteriostatic nature of IONPs-CTAB. Generally, if the MBC/MIC ratio is ≤4, the material is identified as a bactericidal agent, while, if the ratio is >4, the material is identified as a bacteriostatic agent [48]. As revealed by the data presented in Table 1, IONPs-CTAB showed an MBC/MIC ratio equivalent to 4, and this indicated their bactericidal activity against all tested bacteria. This bactericidal activity of IONPs-CTAB is likely to reduce the incidence of developing bacterial resistance [49].

#### 2.2.2. Time–Kill Curve Study

**Figure 2:**
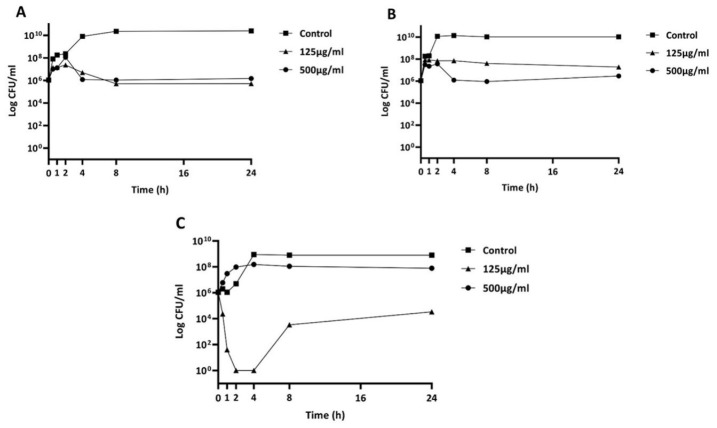
Time–kill curve study of IONPs-CTAB at 125 µg/mL and 500 µg/mL against (**A**) *S. aureus*, (**B**) MRSA, and (**C**) *E. coli*.

To further investigate the antibacterial activity of IONPs-CTAB, a time–kill curve study was conducted against tested bacteria, and the data are presented in Figure 2. Generally, a time–kill curve study is commonly performed using 0.5 to 4 times the MIC value [50,51,52,53,54]. As presented in Table 1, the MIC values of IONPs-CTAB recorded against tested bacteria ranged from 125 to 250 µg/mL, in addition to MBC values that ranged from 500 to 1000 µg/mL. We previously reported [38] that an IONP-CTAB concentration ≥ 250 µg/mL was associated with massive aggregation, and therefore was not recommended to be applied as a antimicrobial agent. This provoked us to test the compatibility of IONPs-CTAB with the eyes and skin of rabbits at a concentration of 250 µg/mL. This concentration proved to be associated with moderate toxicity on abraded skin and eyes of rabbits, and this will be discussed in more detail later in this study.

We previously reported that IONPs-CTAB at a concentration of 125 µg/mL were compatible with the skin and eyes of rabbits [38]. Based on this, IONPs-CTAB at a concentration of 125 µg/mL could be considered as the maximum safe concentration to be used as an antimicrobial agent. However, a time–kill curve study was conducted at a concentration of 125 and 500 µg/mL to tackle the antibacterial behavior of IONPs-CTAB and understand if an increase in IONP-CTAB concentration could be associated with any improvement in antibacterial activity. It was not recommended to go for a higher concentration of IONPs-CTAB due to the incidence of massive particle aggregation that we previously reported [38].

The results revealed that treating *S. aureus* (Figure 2A) and MRSA (Figure 2B) with IONPs-CTAB at concentration of 125 µg/mL resulted in approximately a 4 and 3 log CFU/mL reduction in bacterial count, respectively, compared to untreated bacterial culture (control bacteria in absence of IONPs-CTAB), and this reduction continued to the end of the incubation period. On the other hand, by increasing the concentration of IONPs-CTAB to 500 µg/mL, no marked improvement in antibacterial activity was observed over those recorded at 125 µg/mL (Figure 2A,B).

On the other hand, incubating *E. coli* with IONPs-CTAB at a concentration of 125 µg/mL resulted in a massive drop of CFU/mL by more than 8 log CFU/mL compared to control bacteria at 2 h post-treatment, lasting until 4 h post-treatment (Figure 2C). Interestingly, after 8 h post-treatment, a reduction by more than 5 log CFU/mL was observed compared to the control bacteria, lasting up to 24 h. In contrast, at a concentration of 500 µg/mL, there was a non-significant reduction (*p* > 0.05) in log CFU/mL compared to the control bacteria. Based on this data, the time–kill curve study revealed that IONPs-CTAB at 125 µg/mL can effectively reduce bacterial growth by 5, 4 and 3 log CFU/mL for *E. coli*, *S. aureus* and MRSA, respectively, when compared to the control bacteria.

To the best of the authors’ knowledge, very limited publications [55,56] have investigated the antibacterial activity of IONPs following a time–kill curve study. Huang and colleagues [56], conducted a time–kill curve study for IONPs incorporated into heme (Heme@IONPs) against *S. aureus* persister cells. As revealed from the results, following the time–kill curve study, the bacterial growth inhibition was concentration-dependent, which is inconsistent with what was obtained in our study. However, at MIC (32 µg/mL), only a 1 log CFU/mL reduction in bacterial growth was observed after 5 h post-treatment, which lasted until the end of the incubation (24 h). Notably, this reduction in the bacterial population at the MIC of Heme@IONPs was very low compared with what was recorded in our study. Increasing the concentration of Heme@IONPs to 64 µg/mL (2×MIC) was associated with a 2.7 log CFU/mL reduction in the bacterial population, which was observed at 4 h post-treatment as compared to untreated bacteria. In addition, a complete bacterial reduction was observed at 128 µg/mL (4×MIC). In their study, the MIC value for Heme@IONPs and IONPs was also assessed via the shake flask technique (similar to methodology applied for time–kill curve study), which involved an overnight incubation of tested bacteria with different concentrations of Heme@IONPs and IONPs under shaking conditions followed by serial dilution and plate counting. Following the same technique for investigating antibacterial activity could be used as a confirmatory tool for the obtained results to avoid any confusion that could result from performing different methodologies. In their study [56], the MIC values recorded for Heme@IONPs and IONPs were 32 and 256 µg/mL, respectively. The superior activity of Heme@IONPs over IONPs was linked to their ability to return *S. aureus* persister cells back to their normal metabolic state. This mechanism involved heme-mediated metabolic reactivation, which results in H_2_O_2_ release. This is followed by ^⋅^OH production through an Fe^2+^-mediated Fenton reaction. The authors called this strategy a “revival-killing” process, which involves switching the dormancy state of persister cells and getting them back to normal metabolic conditions, followed by a killing action through the production of ROS [56].

Previously, we reported a time–kill curve study for green-synthesized IONPs against *S. aureus* and *E. coli* [55]. Incubation with green-synthesized IONPs at MBC (200 µg/mL, equivalent to 2×MIC) resulted in a reduction in cell density of both types of bacteria by half at 2 h of incubation, and a complete eradication of bacteria (both types) was observed at 4 h of incubation, indicating the bactericidal effect of these nanoparticles.

In the study of Khan and his colleagues [57], silver nanoparticles were green-synthesized using *Syzygium polyanthum* (SP-AgNPs), with an average particle size of 27.69 nm (measured by TEM), and tested for their antibacterial activity against selected foodborne pathogens, namely *B. cereus*, *B. megaterium*, *B. pumilus*, *B. subtilis*, *E. coli*, *K. pneumoniae*, *L. monocytogenes*, *P. aeruginosa*, *P. mirabilis*, *S. aureus* (ATCC 29737) and *S.* Typhimurium. In their study, SP-AgNPs at their MIC showed a reduction in the bacterial count ranging from 1 to 3 log CFU/mL with all tested pathogens after 4 h post-treatment [57]. These SP-AgNPs showed a pattern of time- and dose-dependent antibacterial activity, where, by increasing the concentration of SP-AgNPs to 2×MIC and 4×MIC, an increase in bacterial growth reduction was observed with complete eradication of the bacterial population of all tested pathogens except for *E. coli*, *K. pneumoniae* and *S*. Typhimurium. For these latter pathogens, increasing the concentration of SP-AgNPs to 4×MIC resulted in a reduction in bacterial growth by only about 3 log CFU/mL after 4 h post-treatment. However, the authors describe SP-AgNPs as a promising eco-friendly and economic antibacterial agent. In addition, the antibacterial activity of SP-AgNPs was traced back to both a disruption of cell wall integrity due to physical damage and destroying the stability and functionality of essential macromolecules due to ROS release.

In another study [58], Ag-NPs were successfully synthesized using bacterial biofilm, where their particle size ranged from 20 to 60 nm, with a zeta potential of −19.1 mV. These NPs demonstrated a remarkable antibacterial activity against *E. coli*, *B. cereus*, and *S. aureus* at their MIC, effectively inhibiting bacterial growth up to 11 h. Furthermore, a concentration lower than the MIC showed a transit inhibitory effect but still effectively suppressing bacterial growth [58]. This behavior was linked to the adhesion of nanoparticles to the cell membrane, resulting in a disruption of the selective permeability of bacteria, leading to the release of cellular contents.

A study conducted by Haque and his colleagues [59] reported that *E. coli* incubated with Ag-NPs at their MIC value revealed a reduction in bacterial growth for 12 h that was followed by a re-growth of bacterial cells. The latter was linked to increasing the lag time of bacterial growth instead of suppressing bacterial growth. The time required for bacterial generation is one of the main characteristic features that contributes to bacterial infectivity; thus, delaying the time at which bacteria start to duplicate their population through increasing the time of the lag phase might be considered as a good target to fight viable infection [49]. This could explain the behavior of *E. coli* incubated with IONPs-CTAB at 125 µg/mL (sub-MIC), where a massive reduction in log CFU/mL was observed after 2 h and up to 4 h post-incubation, followed by a re-growth of bacterial cells.

From the above, it is clear that the majority of data reported for time–kill curve studies of metal-based nanoparticles with bacteria showed time- and dose-dependent antibacterial activity [57,60], which is inconsistent with the data obtained in our study; as mentioned before, IONPs-CTAB at a concentration ≥250 µg/mL showed a massive aggregation [38], and this could be the reason behind the lower antibacterial activity of IONPs-CTAB observed at 500 µg/mL. Furthermore, in the current study, a variation in the antibacterial activity of IONPs-CTAB was observed. This discrepancy may be attributed to the different methodologies applied for determining MIC/MBC and for the time–kill curve study, which were a microdilution method in 96-well plates and in Erlenmeyer flasks, respectively. The literature has reported that bacterial growth patterns can vary depending on the culture system, as factors such as the volume of medium, oxygen content and inoculum size may significantly influence bacterial growth and consequently the observed antibacterial activity of an active drug [61]. Although the determination of CFU/mL over time is a labor-intensive and time-consuming methodology [62], it remains the most reliable method for dynamically monitoring the bacterial death rate induced by active nanoparticles [63].

Based on this data, IONPs-CTAB at a concentration of 125 µg/mL demonstrated an effective antibacterial activity against both Gram-positive and Gram-negative bacteria tested in the current study. In addition, a four-times increase in IONPs-CTAB (500 µg/mL) was not associated with any marked improvement of antibacterial activity. Furthermore, we previously reported the biocompatibility and safety of IONPs-CTAB at 125 µg/mL [38]. Therefore, this concentration will be further applied to in vivo studies for therapeutic management of non-infected and infected wounds.

#### 2.2.3. TEM Study

**Figure 3:**
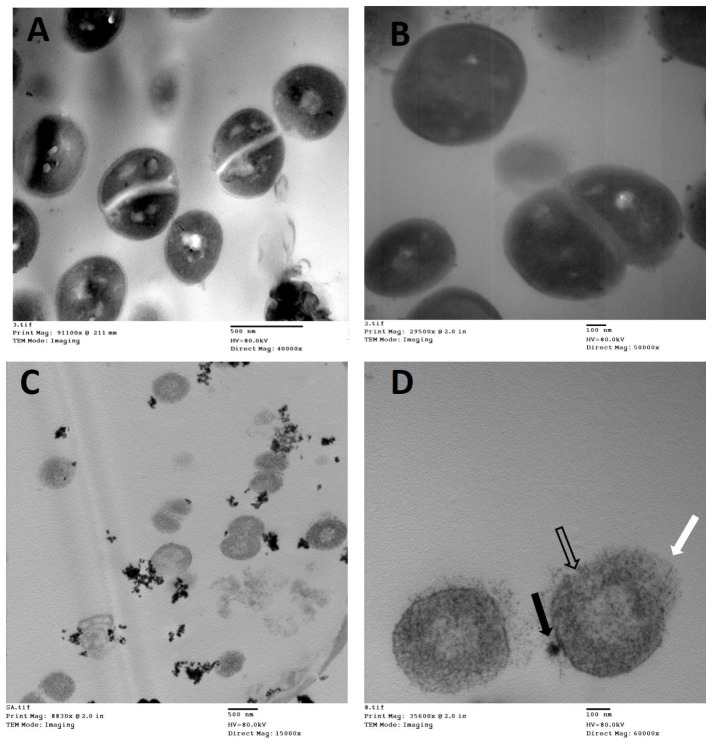
TEM images illustrating the ultrastructural changes in *S. aureus* cells following overnight incubation with IONPs-CTAB at concentration of 125 µg/mL. Images (**A**,**B**) show fields of untreated bacterial cells with no structural changes, as indicated by intact cell wall, and normal intracellular components. Images (**C**,**D**) show structural abnormalities after treatment with IONPs-CTAB. Image (**C**) shows an overview of the field where cells appeared abnormal, with overall distribution of IONPs-CTAB clusters. Image (**D**) shows destructed cells with ruptured cell wall (hollow arrow) and cytoplasmic leakage (white arrow), with nanoparticles distributed on treated bacterial surface (black arrow).

**Figure 4:**
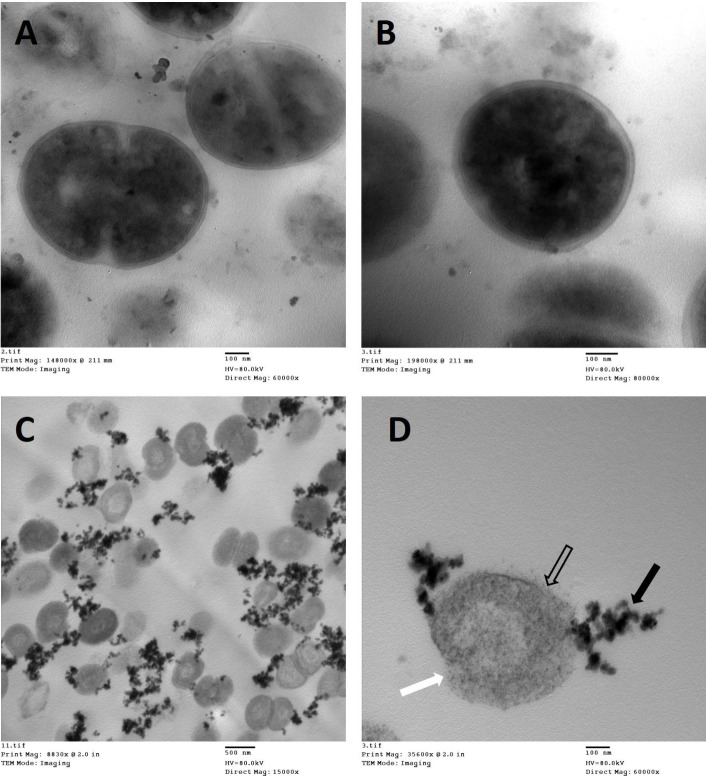
TEM images illustrating the ultrastructural changes in MRSA cells following overnight incubation with IONPs-CTAB at concentration of 125 µg/mL. Images (**A**,**B**) show fields of untreated bacterial cells with no structural changes, as indicated by intact cell wall, and normal intracellular components. Images (**C**,**D**) show structural abnormalities after treatment with IONPs-CTAB. Image (**C**) shows an overview of the field where cells appeared abnormal, with overall distribution of IONPs-CTAB clusters. Image (**D**) shows destructed cells with ruptured cell wall (hollow arrow) and cytoplasmic leakage (white arrow), with nanoparticles distributed on treated bacterial surface (black arrow).

**Figure 5:**
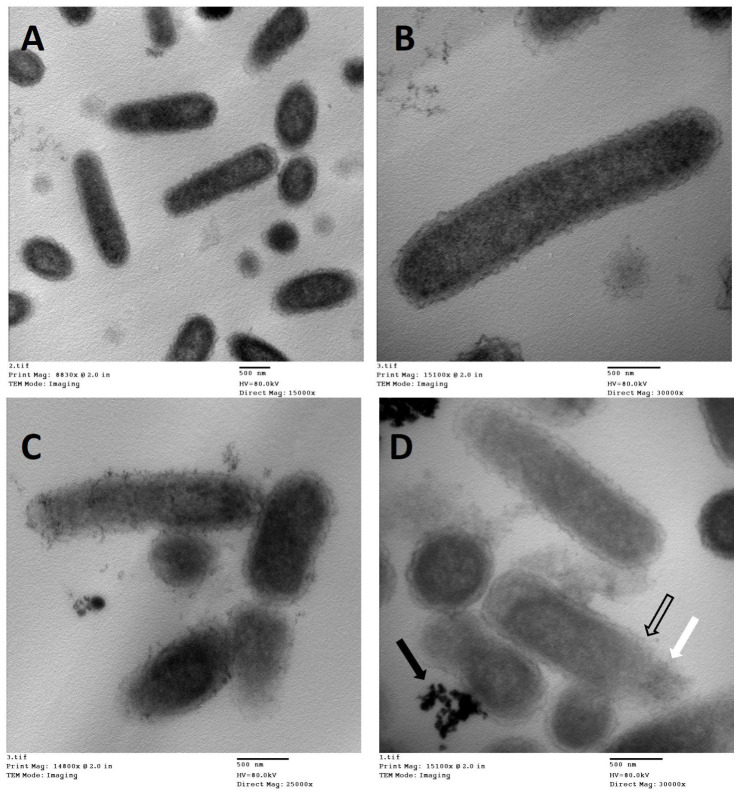
TEM images illustrating the ultrastructural changes in *E. coli* cells following overnight incubation with IONPs-CTAB at concentration of 125 µg/mL. Images (**A**,**B**) show fields of untreated bacterial cells with no structural changes, as indicated by intact cell wall, and normal intracellular components. Images (**C**,**D**) show structural abnormalities after treatment with IONPs-CTAB. Image (**C**) shows an overview of the field where cells appeared abnormal, with IONPs-CTAB clusters. Image (**D**) shows destructed cells with ruptured cell wall (hollow arrow) and cytoplasmic leakage (white arrow), with nanoparticles distributed on treated bacterial surface (black arrow).

To further investigate the antibacterial activity mechanisms of IONPs-CTAB at a concentration of 125 µg/mL against *S. aureus*, MRSA and *E. coli*, a TEM study was conducted to elucidate the ultrastructural differences between treated and untreated bacterial cells. The obtained TEM images of *S. aureus*, MRSA and *E. coli* are presented in Figure 3, Figure 4 and Figure 5, respectively.

As revealed in Figure 3, untreated cells of *S. aureus* exhibit normal shape with no abnormal structural changes (Figure 3A), where cells appear with intact cell walls and normal cell content (Figure 3B). However, *S. aureus* cells treated with IONPs-CTAB at 125 µg/mL showed abnormal appearance, where IONP-CTAB clusters appeared clearly in the field (Figure 3C). This abnormality included ruptured cell walls (Figure 3D, hollow arrow) with cytoplasmic leakage (Figure 3D, white arrow) and IONPs-CTAB adhering to bacterial cell walls (Figure 3D, black arrow).

Similarly, cells of MRSA treated with IONPs-CTAB showed structural changes compared to untreated cells. The TEM images showed a normal appearance of untreated cells (Figure 4A), where cells showed complete un-itched cell walls and normal cell contents (Figure 4B). Upon treating MRSA cells with IONPs-CTAB at a concentration of 125 µg/mL, structural abnormalities were observed (Figure 4C). These abnormalities were represented as detached cell walls (Figure 4D, hollow arrow) and the escaping of cytoplasmic content (Figure 4D, white arrow), with observed aggregations of IONPs-CTAB on bacterial cell walls (Figure 4D, black arrow).

On the other hand, the TEM images of untreated *E. coli* cells showed a normal structure (Figure 5A), as cells appeared with complete cell walls and maintained intracellular components (Figure 5B). However, IONP-CTAB (125 µg/mL)-treated cells showed structural changes (Figure 5C) represented by deteriorated cell walls (Figure 5D, hollow arrow) and the leakage of cytoplasmic content (Figure 5D, white arrow), and, again, IONPs-CTAB adhered to bacterial cell walls.

Different mechanisms may contribute to the antibacterial activity of IONPs; however, ROS release and/or physical damage are the most reported mechanisms [38,55,64,65], where released ROS damages bacterial macromolecules including DNA, proteins and lipids, which results in great oxidative injury and eventually bacterial death [38,66,67]. In our previous publication [38], we proved that concentration-dependent oxidative stress was one of the possible mechanisms by which IONPs-CTAB exert their antibacterial activity against clinical isolates of *S. aureus* and *E. coli*. This was confirmed by the disrupted bacterial antioxidant machinery of the tested isolates. Moreover, bacterial surfaces are negatively charged, where IONPs can adhere physically to their walls via electrostatic and van der Waals interactions [55,68,69,70,71]. As a result, clusters of IONPs are formed on bacterial cell walls, which leads to a sequence of events including the disruption of cell wall integrity, increasing cell membrane permeabilization, and leakage of intracellular contents [55,60,72]. This sequence of events is consistent with what was observed in the TEM images obtained in the current study. In our more recent publication [55], green-synthesized IONPs stabilized with tween 80 (IONPs-GTw80) showed great antibacterial activity against isolates of *S. aureus* and *E. coli*, and TEM images revealed an accumulation of IONPs-GTw80 on the surface of both tested bacteria.

As a result, we concluded that physical damage may be one of the mechanisms contributing to the antibacterial activity of IONPs-GTw80 [55]. The latter is consistent with the adherence of IONPs-CTAB observed with bacterial isolates tested in the current study. Collectively, the antibacterial activity of IONPs-CTAB might be linked to both ROS release and physical damage.

### 2.3. In Vivo Studies

#### 2.3.1. Irritation Test

##### Skin Irritation Test

**Figure 6:**
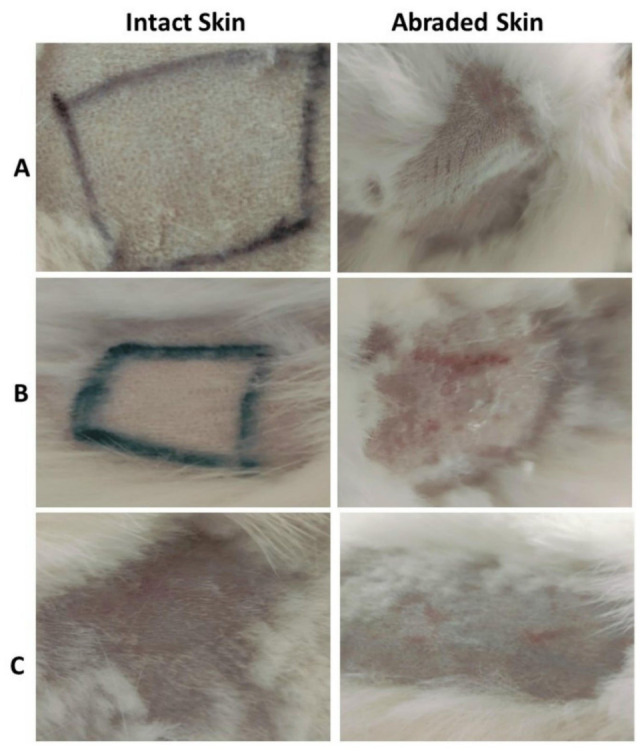
Representative photos of the skin irritation test for one representative rabbit for (**A**) untreated group [55], (**B**) IONP-CTAB (250 µg/mL)-treated group and (**C**) blank-treated group. For each treatment, photographs represent both intact skin (normal uninjured skin) and abraded skin (superficially scratched skin with disrupted stratum corneum without bleeding). No differences in score were observed after 72 h between the test and control sites for both abraded and intact skin except for the IONP-CTAB (250 µg/mL)-treated group, where a moderate inflammatory reaction was observed in treated abraded sites only.

Table 2 and Figure 6 present the obtained data on treating both intact and abraded rabbit skin with IONPs-CTAB at a concentration of 250 µg/mL. As revealed, a noticeable erythema and inflammation were observed after 24 and 72 h in abraded skin treated with IONPs-CTAB (250 µg/mL). Contrary to this, no sign of irritation was observed for intact skin, as with the untreated control site, for all investigated parameters, including erythema, eschar, and edema. However, the untreated and blank groups revealed an initial and temporal erythema (lasting only for 30 min) with the abraded skin, and this was linked to needle abrasion.

##### Eye Irritation Test

**Figure 7:**
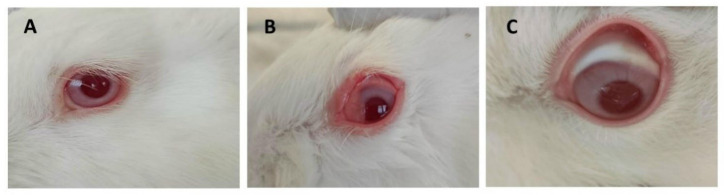
Representative photos of the eye irritation test for one representative rabbit for (**A**) untreated, (**B**) treated with IONPs-CTAB (250 µg/mL) and (**C**) blank-treated group. The cornea, iris, and conjunctiva were observed after 72 h. No differences were observed in the cornea, iris, or conjunctiva between the untreated and blank groups. However, the IONP-CTAB-treated group (250 µg/mL) showed mild to moderate redness of the cornea and conjunctiva.

The ocular irritation assessment, illustrated in Table 3 and Figure 7, indicated that both the untreated and blank solution exhibited normal iridial, corneal, and conjunctival conditions, with no discernible abnormalities. This is in contrast with eyes treated with IONPs-CTAB at a dose of 250 µg/mL, where a mild to severe erythema of the conjunctiva and cornea, accompanied by global ocular hyperemia, was observed.

Both untreated and Blank groups revealed no signs of any eye irritation; thus, they were both given a score of 0. Moderate signs of eye irritation were observed in the animal group treated with IONPs-CTAB (250 µg/mL). During the observations, attention was paid to eye discharge, conjunctival edema, how responsive the iris was, and corneal opacity.

##### Histopathological Evaluations

**Figure 8:**
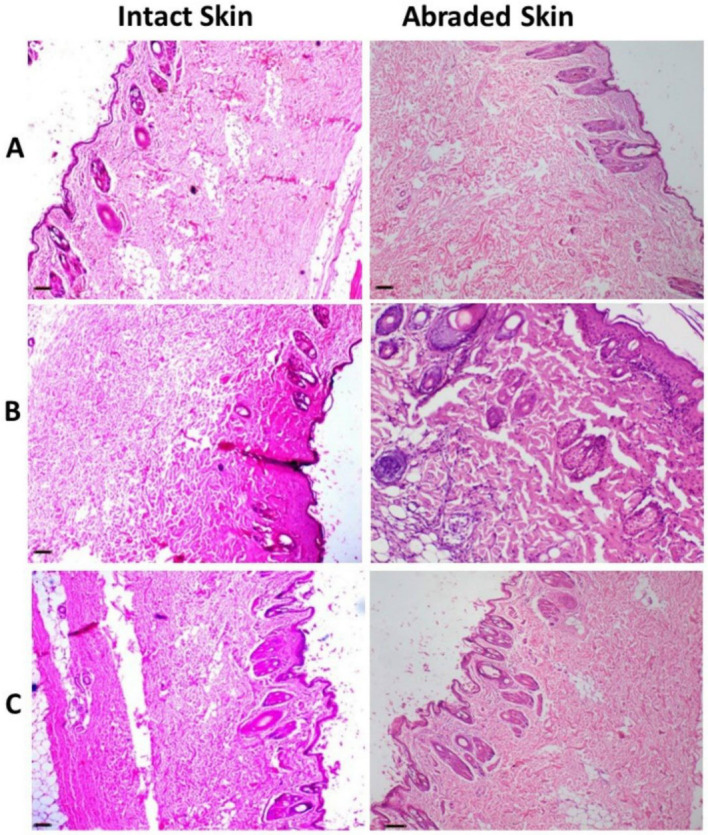
Representative H&E-stained photomicrographs of skin sections from rabbits indicated that the intact skin displayed normal histological architecture across all groups, including (**A**) untreated [55], (**B**) IONP-CTAB-treated (250 µg/mL) and (**C**) blank-treated groups, with no signs of erosion, ulceration, necrosis, or inflammatory cell infiltration after 72 h. Likewise, the abraded skin in the untreated and blank groups exhibited normal histological characteristics. Conversely, abraded skin treated with IONPs-CTAB at 250 µg/mL exhibited histopathological changes marked by inflammatory cell infiltration in both the epidermal and dermal layers, accompanied by dilated blood vessels and indications of inflammation, signifying a localized inflammatory response at this concentration (magnification power 100×).

The histopathological results of the skin irritation test for both intact and abraded skin are presented in Figure 8. Histopathological analysis demonstrated that the intact skin maintained normal architecture devoid of any signs of inflammation in all tested animals, encompassing untreated, IONP-CTAB, and blank-treated groups. In contrast, abraded skin treated with IONPs-CTAB at a dose of 250 µg/mL exhibited distinct signs of inflammation, marked by the infiltration of inflammatory cells in both the epidermal and dermal layers, as well as dilated blood vessels and concomitant inflammatory alterations.

**Figure 9:**
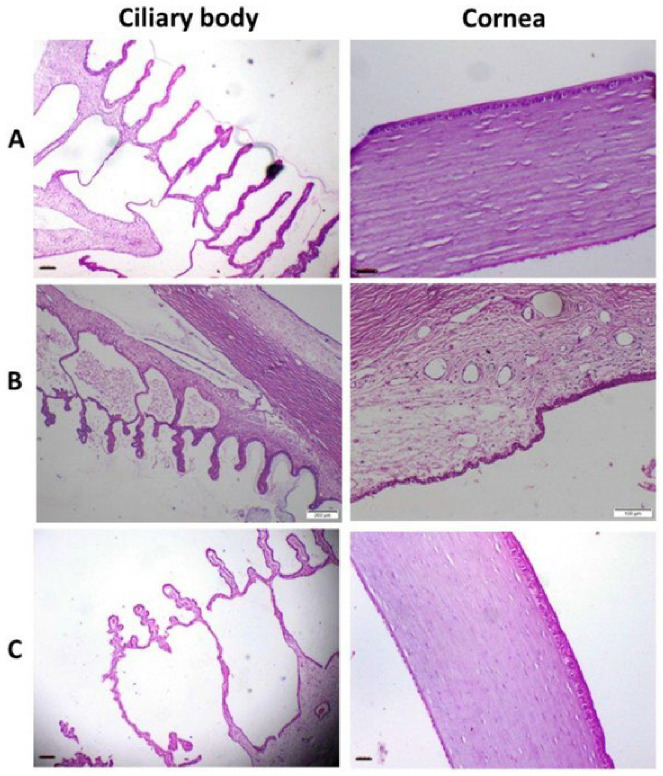
Representative H&E-stained photomicrography from the eye irritation test for (**A**) untreated group [55], (**B**) IONP-CTAB (250 µg/mL)-treated group and (**C**) blank-treated group. Images indicate that both the untreated and blank-treated animals displayed a normal ocular appearance, with no discernible changes in the cornea, iris, or conjunctiva after 72 h. Conversely, eyes treated with IONPs-CTAB at a dose of 250 µg/mL (**B**) exhibited pronounced ocular irritation, marked by conjunctival, iridal, and corneal erythema along with inflammatory alterations, signifying mild to moderate ocular inflammation (magnification power 100×).

Histopathological examination of the eye, as depicted in Figure 9, demonstrated normal histological architecture of the cornea, fibrous connective tissue, and ciliary body in both the untreated and blank-treated animals, with no indications of inflammation, erosion, ulceration, or necrobiotic alterations. Conversely, eyes treated with IONPs-CTAB at a dose of 250 µg/mL had pronounced pathological changes, including ocular irritation characterized by conjunctival and corneal erythema, inflammatory responses, and indications of tissue reactivity. Histological sections exhibited inflammatory reactions characterized by vascular congestion and inflammatory cell infiltration, indicating irritation and inflammation at the tested concentration of IONPs-CTAB (250 µg/mL). These results indicated that, while the blank solution and untreated eyes were compatible with ocular tissues, IONPs-CTAB at 250 µg/mL elicited discernible ocular inflammatory alterations.

The inflammatory responses noted in both ocular and dermal tissues at the elevated concentration of IONPs-CTAB (250 µg/mL), compared to absence of any sign of inflammation we previously reported at 125 µg/mL, can be ascribed to concentration-dependent nano-toxic effects. Previously, we reported that IONPs-CTAB produce concentration-dependent ROS [38], which leads to oxidative stress and the activation of inflammatory signaling pathways like NF-κB and MAPK [73,74]. When the ROS amount increases, they can overwhelm the body’s natural defenses; this might cause serious adverse effects such as the blockage of blood vessels, infiltration of inflammatory cells into tissues, and irritation of tissues, especially in sensitive areas like the eye and broken skin [75]. Additionally, the presence of CTAB on the nanoparticle surface may enhance their contact with the cell membrane followed by cellular entry, causing local inflammatory reactions that are reported to be serious at large doses [76]. The integrity of intact skin presumably restricted nanoparticle penetration, elucidating the lack of histopathological alterations in intact skin across all groups, while abraded skin demonstrated heightened vulnerability to inflammation [77]. Therefore, IONPs-CTAB at 250 µg/mL are unsuitable for the management of wound healing.

Based on the obtained data, IONPs-CTAB at 125 µg/mL were proven to control bacterial growth and demonstrated to be compatible with skin (intact/abraded) and eyes, as we previously reported [38]. Thus, it is highly recommended to investigate the wound healing activity of IONPs-CTAB at a concentration of 125 µg/mL.

##### Wound Healing Activity Study

**Figure 10:**
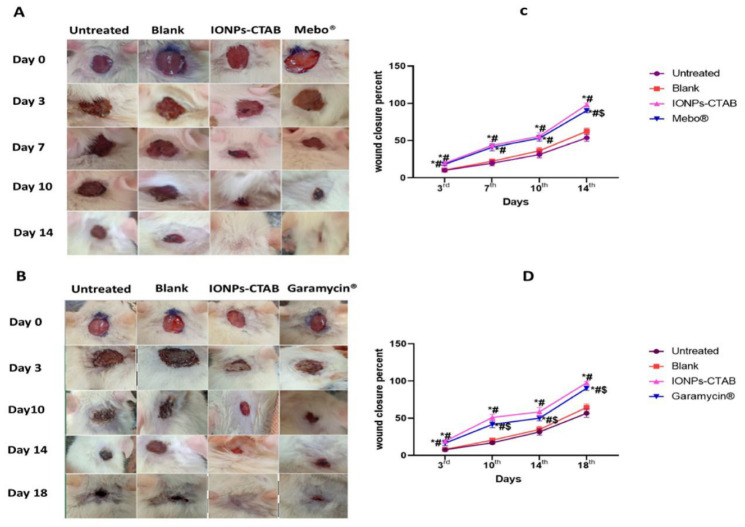
Representative images of wound closure for (**A**) non-infected and (**B**) infected wounds in animals, captured at predetermined time intervals for both untreated group and groups treated with blank, IONPs-CTAB (40 µL, 125 µg/mL), and standard treatment: Mebo^®^ ointment and Garamycin^®^ cream. Wound closure percentage recorded for (**C**) non-infected and (**D**) infected wounds of untreated and treated animals. The graph shows results as a mean value ± SD, *n* = 6. At *p* < 0.05, significant differences from untreated, blank, and IONPs-CTAB were found to be *, #, and $, respectively.

The wound healing activity of IONPs-CTAB (40 µL, 125 µg/mL) for both non-infected and *S. aureus*-infected wounds was assessed in vivo against standard treatment—MEBO^®^ ointment and Garamycin^®^ cream, respectively—and the obtained data are presented in Figure 10. MEBO^®^ ointment was reported as the standard treatment for the management of non-infected wounds [3,78,79], while Garamycin^®^ cream (Gentamicin) was applied as a standard treatment for the management of infected wounds, based on the antibiotic susceptibility test performed for the *S. aureus* bacterial isolate used in the current study (Table S1, Supplementary Materials).

No slough formation was observed in the non-infected wound (Figure 10A); instead, the wound bed began to dry out on the edges on day 3, resulting in the formation of eschar. In contrast, slough development in the wound bed of the infected wounds was initially observed on day 3 (Figure 10B) and started to resolve by day 10 in the IONP-CTAB-treated group.

Topical application of IONPs-CTAB (40 µL) at a dose of 125 µg/mL promoted wound healing in both non-infected and infected groups, as revealed by a significant (*p* < 0.05) increase in the wound closure percentage, which was 82.4%, and 72.4% at day 14 (Figure 10C) and day 18 (Figure 10D) post-treatment, respectively, when compared to the untreated group. By comparing the wound healing activity of IONPs-CTAB at 125 µg/mL to the standard treatment, a significant (*p* < 0.05) increase in wound closure percentage, equivalent to 8.7% and 8% on day 14 and day 18, was recorded over standard therapeutic treatment applied for non-infected and infected wounds, respectively. The superior wound healing observed with IONPs-CTAB for both non-infected and infected wounds compared to standard treatment might be linked to an increase in collagen expression and epithelial thickness [78,79]. However, as expected, infected wounds required a longer healing time than non-infected wounds, which is consistent with the literature [7,34].

##### Histopathological Examination

**Figure 11:**
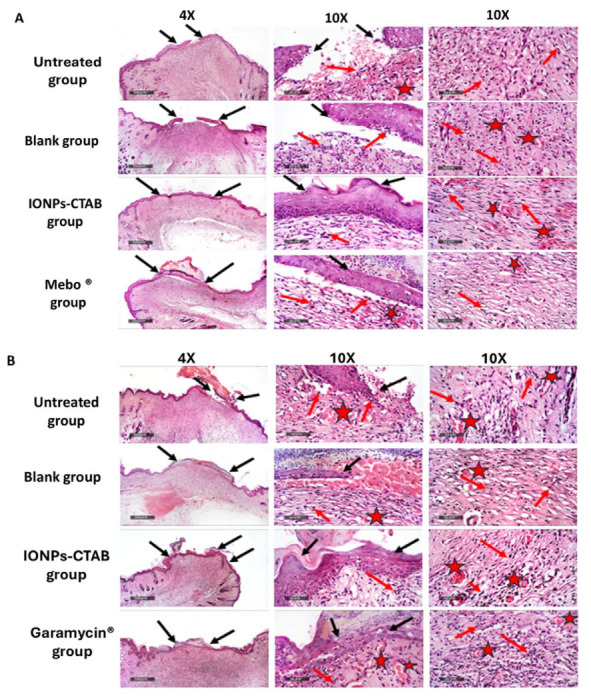
Representative histological image of skin layers in various groups. (**A**) Non-infected skin tissue collected on day 14 of the experiment and (**B**) infected skin tissue collected on day 18 of the experiment for both untreated group and groups treated with blank, IONPs-CTAB (40 µL, 125 µg/mL), and standard treatment: Mebo^®^ ointment and Garamycin^®^ cream. Black arrow: wound gap; red arrow: aggregates of inflammatory cells; red star: blood vascularity. Scale bar, 4× = 500 and 10× = 50 μm.

Regarding the non-infected wound, as presented in Figure 11A, the untreated group revealed a persistent, narrow open wound gap associated with focal epidermal loss, a blood-filled ulcer, and a depressed area of necrotic tissue (black arrow). Minimal organized collagen fibers were visible throughout the dermal layer, along with moderate mixed inflammatory cell infiltrates (red arrow). The wound gap and lesion recorded for blank samples were identical to those of untreated samples, indicating a lack of protective efficacy. On the other hand, IONPs-CTAB (40 µL, 125 µg/mL) showed a complete wound gap bridging and closure, with a thick epidermal layer (black arrow), as well as an obvious increased dermal fibroblastic activity and more ordered dermal collagen fibers. There was a slight infiltration of focal inflammatory cells into the dermal layers (red arrow). Nonetheless, hyperemic dermal vasculature with frequent blood extravasation was observed (red star). Concerning standard treatment (Mebo^®^ ointment), complete epithelialization was observed beneath a thick, bloody scab mixed with necrotic tissue, resulting in a depression and separation of the epidermal layers from the underlying dermis (black arrow). Moderate fibroblastic activity was correlated with a moderate presence of mature collagen fibers; however, persistent hydremic blood vessels (red star) and moderate subepithelial dermal inflammatory cell infiltration (red arrow) were observed.

Concerning infected wounds, as presented in Figure 11B, the untreated group revealed a persistent wound gap with focal epidermal loss and ulceration, covered in a hemorrhagic scab with necrotic tissue depression (black arrow). The dermal layer showed numerous infiltrations of inflammatory cells (red arrow), congested blood vessels (red star), and a few organized collagen bands. The hemorrhagic wound gap and lesion records of the blank sample were identical to those of untreated samples without any indication of protective efficacy. However, IONPs-CTAB displayed a thick epidermal layer with focal parakeratosis (black arrow), whole-wound bridging and closure, and obviously increased dermal fibroblastic activity, with well-organized dermal collagen fibers. Nonetheless, congested blood vessels (red star) and focal inflammatory cell infiltration in the deep dermal layers (red arrow) were noted. Concerning standard treatment (Garamycin^®^ cream), incomplete re-epithelialization of the epidermal layers beneath the scab of necrotic tissue (black arrow) was observed, associated with delayed healing progression. However, persistent dermal inflammatory cell infiltration (red arrow) and numerous hyperemic blood vessels (red star) were observed, with moderate amounts of mature collagen fibers, correlating with moderate fibroblastic activity.

Therefore, based on the obtained results, both non-infected and infected wounds treated with IONPs-CTAB (40 µL, 125 µg/mL), when compared to standard treatment, revealed the best improvement of the histological structure of the skin, with enhanced skin regeneration through an upregulation of fibroblast activity and increased collagen fiber presence, which is consistent with previous studies [7,31,32,35,80].

**Figure 12:**
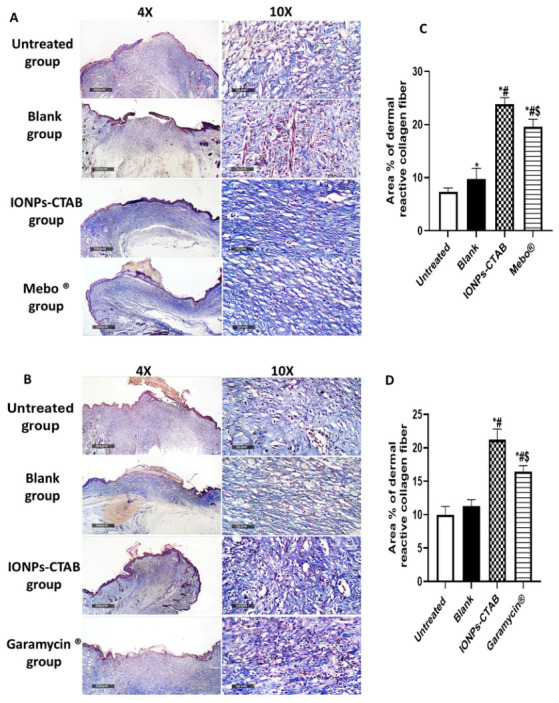
Representative Masson’s trichrome-stained photomicrographs: (**A**) non-infected and (**B**) infected wound. Area % of collagen expression in (**C**) non-infected wound and (**D**) infected wound for both untreated group and groups treated with blank, IONPs-CTAB (40 µL, 125 µg/mL), and standard treatment: Mebo^®^ ointment and Garamycin^®^ cream. The graph shows results as mean ± SD, *n* = 6. At *p* < 0.05, significant differences from the untreated, blank, and IONP-CTAB groups were found to be *, #, and $, respectively. Scale bar, 4× = 500, 10× = 50 μm.

Masson’s trichrome stain was conducted to tackle the regeneration of the wound area and the number of skin appendages and relative collagen deposition in different treated groups, and the results are presented in Figure 12A for non-infected and Figure 12B for infected groups. In the current study, topical application of IONPs-CTAB (40 µL, 125 µg/mL) revealed a significant (*p* < 0.05) increment in the expression of collagen fiber for both non-infected and infected groups by 2.3- and 1.1-fold, respectively, compared to the untreated group (Figure 12C,D). Furthermore, IONPs-CTAB revealed a significant (*p* < 0.05) upregulation of the expression of collagen fiber in both non-infected and infected groups by 21%, and 29%, respectively, compared to standard treatment (Figure 12C,D). The data obtained are consistent with previous studies where the beneficial wound healing activity of iron and the dinitrosyl iron complex in non-infected wounds was linked to its ability to enhance the activity of fibroblast cells as well as collagen expression [81,82]. Furthermore, another study reported the wound healing activity of CuO-NPs that were chemically synthesized, with a particle size of 301.80 ± 9.27 nm and zeta potential values of −5.40 ± 1.09 mV. The application of CuO-NPs was associated with a marked increase in collagen expression, and this promoted wound healing activity in both non-infected and infected wounds in a diabetic animal model [7].

##### Oxidative Stress and Inflammatory Markers

The biological process of wound healing is intricate and includes hemostasis, inflammation, proliferation, and matrix remodeling. ROS are essential for regulating key processes such as angiogenesis, platelet activation, and antimicrobial defense. On the other hand, elevated ROS levels can induce oxidative stress (OS), which can disrupt the healing cascade and lead to inflammation, chronic wounds, and poor tissue repair [83].

IONPs were reported to modulate ROS in two ways: either producing moderate ROS to promote regenerative processes or scavenging excessive ROS to reduce oxidative damage. IONPs’ capacity to tune ROS is essential for preserving redox homeostasis and promoting wound healing. IONPs can mimic catalase and peroxidase, enabling them to scavenge superoxide and H_2_O_2_ radicals and reduce oxidative stress at the wound site [84]. Concurrently, the controlled production of ROS by IONPs promotes angiogenesis, cellular proliferation, and extracellular matrix remodeling, the key events in tissue regeneration. By modulating the redox balance, IONPs also support immune signaling by scavenging excess ROS, thereby minimizing host tissue damage while enhancing antimicrobial defense, which occurs at concentrations ranging from (10 to 100 μg/mL) [31]. These findings align with our study, which found that IONPs-CTAB at a concentration of 250 μg/mL showed a moderate cytotoxic effect on the abraded skin and eyes of rabbits, contrary to absence of any cytotoxicity at a concentration of 125 μg/mL [38].

**Figure 13:**
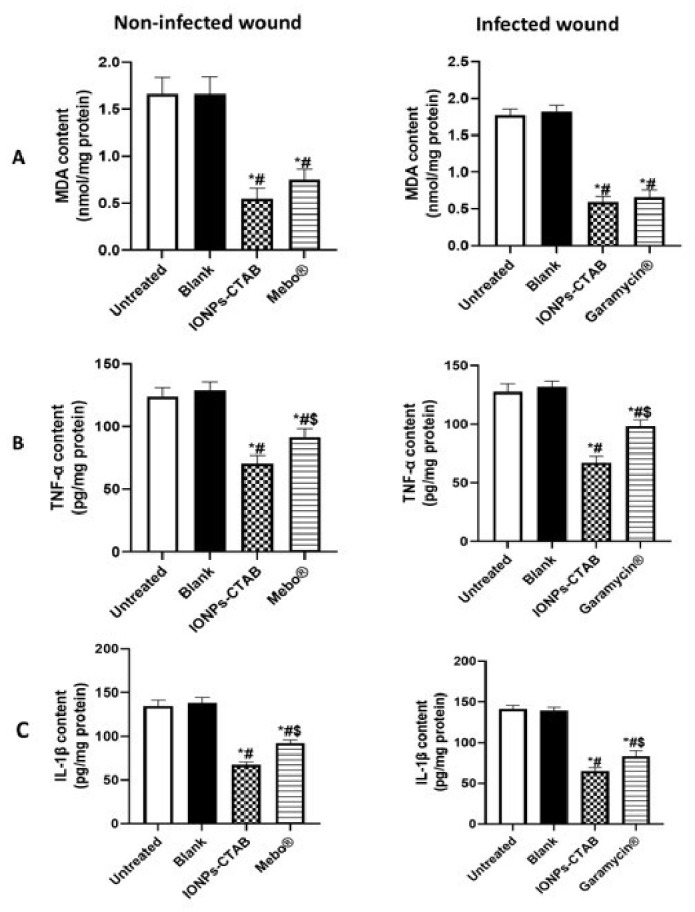
Effect of topical application of IONPs-CTAB on oxidative stress and inflammatory markers in non-infected and infected wound homogenate; (**A**) MDA, (**B**) TNF-α and (**C**) IL-1β content for untreated group and groups treated with blank, IONPs-CTAB (40 µL, 125 µg/mL), and standard treatment: Mebo^®^ ointment and Garamycin^®^ cream. The graph shows results as mean ± SD, *n* = 6. At *p* < 0.05, significant differences from the untreated, blank, and IONP-CTAB groups were found to be *, #, and $, respectively.

To ascertain this, the levels of malondialdehyde (MDA), interleukin- 1β (IL-1β), and tumor necrosis factor-alpha (TNF-α) in tissues were assessed, and the obtained data were presented in Figure 13. As revealed by Figure 13A, there was a significant (*p* < 0.05) reduction in malondialdehyde (MDA) content by 67.1% and 66.4%, respectively, compared to untreated groups. Moreover, it was found that the non-infected and infected group treated with IONPs-CTAB showed a decrease in the content of MDA by 27.4% and 10%, respectively, as compared to standard groups (Mebo^®^ ointment and Garamycin^®^ cream). This is indicative of a downregulation of the oxidative stress in non-infected and infected wounds treated with IONPs-CTAB (40 µL, 125 µg/mL).

Injury-induced signals, including pathogen-associated molecular patterns (PAMPs) from bacterial components such as lipopolysaccharides, and damage-associated molecular patterns (DAMPs) released by necrotic cells and damaged tissue, trigger the immune response. Circulating leucocytes are drawn to the site of injury by the subsequent release of pro-inflammatory cytokines, specifically interleukin-1β (IL-1β) and tumor necrosis factor-alpha (TNF-α) [83]. IONPs were reported to reduce pro-inflammatory cytokines, such as TNF-α and IL-6, which are elevated in chronic wounds, by downregulating NF-κB signaling and mitigating ROS-induced oxidative damage [31]. This is in line with the results obtained in the current study, which found that both non-infected and infected wounds treated with IONPs-CTAB (40 µL, 125 µg/mL) exhibited a significant (*p* < 0.05) decrease in TNF-α content by 43.3%, and 47.5%, respectively (Figure 13B). Meanwhile, compared to their perspective standard therapy, the group treated with IONPs-CTAB showed a significant (*p* < 0.05) decrease in TNF-α content by 23.3% and 31.8% in non-infected and infected groups, respectively (Figure 13B). In addition, an obvious decline in IL-1β content by 50% and 54%, respectively, in comparison to the untreated group was recorded (Figure 13C). Meanwhile, compared to their perspective standard therapy, the group treated with IONPs-CTAB showed a significant (*p* < 0.05) decrease in TNF-α content by 23.3% and 31.8% in non-infected and infected groups, respectively (Figure 13B). Furthermore, a noticeable reduction in IL-1β content by 27% and 21.8% was also observed in the non-infected and infected groups, respectively, as compared to standard therapy (Figure 13C).

##### Expression Levels of VEGF and TGF

Angiogenesis creates new blood vessels from established vasculatures, which are necessary for tissues that are wounded to get immune cells, nutrients, and oxygen. VEGF promotes endothelial cell migration, proliferation, and the formation of capillary tubes. Previous research indicated that IONPs can stimulate angiogenesis by activating pro-angiogenic pathways and increasing VEGF expression [31,85,86]. It has been demonstrated that IONPs induce oxidative stress or mild hypoxic-like conditions, which stabilize hypoxia-inducible factor-1 alpha (HIF-1α), a transcription factor that directly increases VEGF expression [86]. Important intracellular signaling cascades, such as MAPK/ERK and PI3K/Akt, can be triggered by IONPs, leading to increased VEGF transcription and enhanced angiogenic responses in endothelial cells. Research has demonstrated that, through paracrine VEGF signaling, functionalized IONPs can promote endothelial cell migration, proliferation, and tubule formation [31].

**Figure 14:**
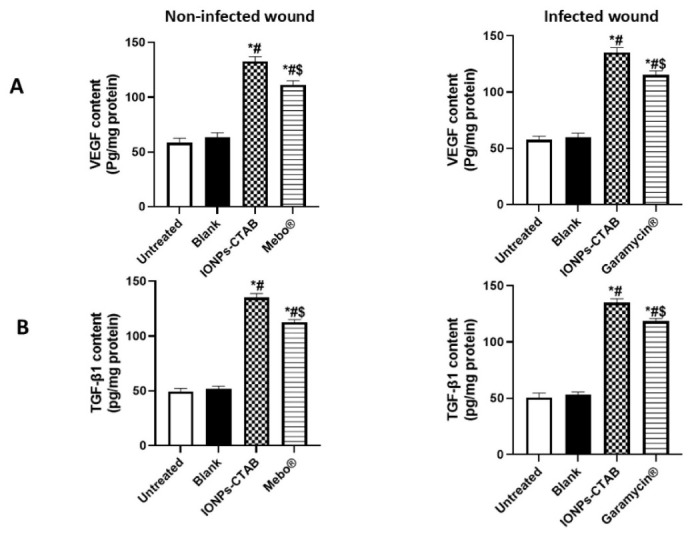
Effect of topical application of IONPs-CTAB on (**A**) VEGF and (**B**) TGF-β1 content in non-infected and infected wound homogenate, for untreated group and groups treated with blank, IONPs-CTAB (40 µL, 125 µg/mL) and standard treatment: Mebo^®^ ointment and Garamycin^®^ cream. The graph shows results as mean ± SD, *n* = 6. At *p* < 0.05, significant differences from the untreated, blank, and IONP-CTAB groups were found to be *, #, and $, respectively.

This is consistent with the findings in the current study, where non-infected and infected wounds treated with IONPs-CTAB displayed a significant (*p* < 0.05) elevation of the VEGF level by 1.27-and 1.34-fold, respectively, compared to the untreated group. Furthermore, non-infected and infected wounds treated with IONPs-CTAB demonstrated a significant (*p* < 0.05) increase in VEGF levels by 19% and 17.2%, respectively, compared to standard treatment (Figure 14A).

According to previous reports, activated platelets in the hemostatic phase secrete transforming growth factor-β1 (TGF-β1), which recruits inflammatory cells, stimulates fibroblast and keratinocyte proliferation and migration, promotes wound contraction, and remodels the extracellular matrix to ensure the last stage of wound healing [7]. Numerous cell types, such as neutrophils, macrophages, fibroblasts, and endothelial cells, are involved in the healing process of cutaneous wounds. Every stage of wound healing depends on TGF-β1, which generally inhibits the inflammatory response and promotes the development of granulation tissue in injured areas [87]. Growth factors like transforming growth factor-beta 1 (TGF-β1), which further promote extracellular matrix (ECM) synthesis and modify fibroblast behavior, are reported to be activated by IONPs [31]. Again, this is consistent with the results obtained in the current study, where the content of TGF-β1 was significantly (*p* < 0.05) upregulated in non-infected and infected wounds treated with IONPs-CTAB by 1.73-and 1.66-fold, respectively, compared to the untreated group. Moreover, non-infected and infected wounds treated with IONPs-CTAB demonstrated a significant (*p* < 0.05) increase in TGF-β1 of 20% and 14%, respectively, compared to standard treatment (Figure 14B).

##### Levels of Hydroxyproline

**Figure 15:**
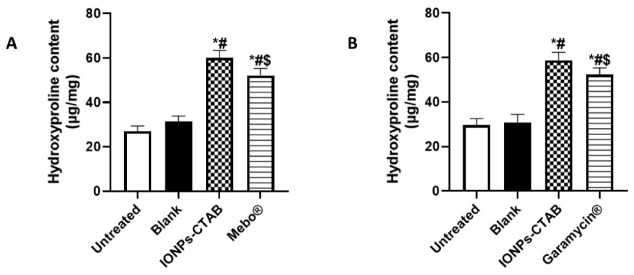
Effect of topical application of IONPs-CTAB on hydroxyproline content: (**A**) non-infected and (**B**) infected wound homogenate for untreated group and groups treated with Blank, IONPs-CTAB (40 µL, 125 µg/mL) and standard treatment: Mebo^®^ ointment and Garamycin^®^ cream. The graph shows results as mean ± SD, *n* = 6. At *p* < 0.05, significant differences from untreated, blank, and IONP-CTAB groups were found to be *, #, and $, respectively.

The remodeling phase is indicated by hydroxyproline, which is thought to be a sign of protein and collagen deposition at the wound site [88]. In the present study, it was documented that non-infected and infected wounds treated with IONPs-CTAB showed a significant (*p* < 0.05) increment in the content of hydroxyproline by 120% and 97.7%, respectively, compared to untreated groups. Furthermore, non-infected and infected wounds treated with IONPs-CTAB demonstrated a significant (*p* < 0.05) increase in hydroxyproline content by 15% and 12%, respectively, compared to standard treatment (Figure 15A,B).

Looking at the literature, limited studies have applied IONPs for the treatment of wounds [34,35,37]. Mandarada and colleagues [35] reported a very high dose of myco-synthesized iron oxide nanoparticles (500 μg/day, i.e., 100 times the dose of IONPs-CTAB applied in the current study) injected intra-peritoneally (i.p.) to achieve successful wound healing and wound closure on day 7 after initiating the treatment. However, in their study, a lack of wound healing activity was recorded with chemically synthesized IONPs at a dose of 500 μg/day. The healing activity observed with myco-synthesized iron oxide nanoparticles was linked mainly to pro-angiogenic mechanisms. Notably, myco-synthesized iron oxide nanoparticles were considered safe at 1–5 mg/kg (i.p.) after 24 h and 7 days, contrary to chemically synthesized counterparts that showed reduced tolerability at 2.5 mg/kg (i.p.). This is contrary to the effective wound healing activity observed for IONPs-CTAB at a very low dose (5 μg/day; 40 μL of 125 μg/mL) after topical application, with successful wound healing recorded at day 14 and day 18 for non-infected and infected wounds, respectively. Furthermore, we previously addressed the biocompatibility of IONPs-CTAB at 125 μg/mL with the skin (abraded/intact) and eyes of rabbits [38].

Another study conducted by Guo and colleagues [37] reported the wound healing activity of photo-thermal iron oxide nanoparticles against *S. aureus*-infected wounds after topical application of NPs at a dose 50 μL/day (1000 μg/mL)—i.e., ten times the dose of IONPs-CTAB used in this study—to achieve a successful wound healing at day 9 after initiating the treatment. However, the wound was associated with scar formation. In the same context, Panigrahi and colleagues [34] reported that hydrogel-encapsulating biosynthesized iron oxide nanoparticles (4 mg/mL) combined with platelet-rich plasma accelerated the incidence of wound closure for non-infected wounds to be at 18 days versus 22 days in controls, yet healing was accompanied by noticeable scarring.

Another study conducted by Abeydeera and colleagues [89] assessed the antibacterial and wound healing activity of an Iron (III)–Tropolone Complex. The complex recorded an MIC equivalent to 2 µg/mL against methicillin-susceptible *S. aureus*, methicillin-resistant *S. aureus* (MRSA), vancomycin-intermediate *S. aureus*, and strains with high-level resistance to mupirocin and fusidate. For wound healing assessment, 50 µL of a 1% Iron (III)–Tropolone Complex solution (equivalent to 66 µg iron) was applied once daily to a 5 mm full-thickness wound infected with *S. aureus* (Xen36; 5 × 10^6^ CFU/mL equivalent to 6.7 log CFU/mL). This resulted in comparable healing to that reported in our study achieved after three days, corresponding to a total iron dose of 198 µg (66 × 3). In contrast, IONPs-CTAB in our study achieved complete wound closure at a daily dose of 5 µg for 14 days in non-infected wounds and 18 days in infected wounds, each measuring 8 mm full thickness. This corresponds to a total iron dose of 70 to 90 µg, representing less than 2.8-fold (non-infected) and 2.2-fold (infected) the iron dose required for the Iron (III)–Tropolone Complex dose to achieve comparable healing outcomes. Furthermore, the study conducted by Abeydeera and colleagues did not include any histopathological examination or biochemical analysis, leaving the anti-inflammatory effects of their formulation uncharacterized.

Collectively, despite faster closure rates in some previous studies, these outcomes were achieved using a substantially very high dose of IONPs and/or complex formulations and were often associated with scar formation. In contrast, our study highlights that IONPs-CTAB can achieve successful wound healing at a markedly very low dose, with favorable safety and biocompatibility and lack of any sign of scar formation, underscoring their potential as a more practical and safe therapeutic strategy for the management of both non-infected and infected wounds. However, further studies evaluating long-term safety application and monitoring the potential of their accumulation in the systemic circulation and various organs are still essential for their clinical translation.

## 3. Materials and Methodology

### 3.1. Materials

Ferric chloride anhydrous (purity 98%), Ammonium Hydroxide 30%, and cetyltrimethylammonium bromide (CTAB, purity 99%) were purchased from Oxford reagents; (North Gare, Seaton Carew, Hartlepool, United Kingdom), LOBA CHEMIE; (Colaba, Mumbai, India) and Sigma Aldrich; (St. Louis, MO, USA), respectively. Microbiological media including Muller–Hinton broth (MHB), Muller–Hinton agar (MHA), and tryptone soy broth (TSB) were purchased from Hi-Media, Mumbai, India, as dehydrated and ready to use. Peptone and sodium chloride were collected from Oxoid, Hampshire, UK. Dimethyl sulfoxide (DMSO), and the reagent of 3-(4,5-Dimethylthiazol-2-yl)-2,5-Diphenyltetrazolium Bromide (MTT) were purchased from Honeywell™, Charlotte, NC, USA, and Serva, Heidelberg, Germany, respectively. Phosphate-buffered saline (PBS) tablets were obtained from Merck, Darmstadt, Germany. All chemicals and reagents used in this work were of analytical grade.

Three bacterial strains, *Staphylococcus aureus* (*S. aureus*), MRSA, and *Escherichia coli* (*E. coli*), isolated from infected wounds were collected from a clinical setting in Cairo, Egypt. An antibiotic susceptibility test was conducted for each bacterium, and the results obtained are presented in Table S1 in the Supplementary Materials. The data obtained demonstrated that all isolates were MDR-bacteria.

### 3.2. Methodology

#### 3.2.1. Synthesis and Characterization of IONPs/IONPs-CTAB by Co-Precipitation Method

IONP and IONP-CTAB synthesis and characterization were reported in our previously published protocol [38]. In the current work, a new batch of each were synthesized and characterized using FTIR, DSC-TGA, and XRD analysis to ascertain their successful synthesis. Further analysis was also conducted to determine the particle size, polydispersity index and zeta potential of the new batch using Malvern Zeta-sizer Nano ZS (Malvern Instruments Ltd., Malvern, UK). Morphological examination of the new batch was also assessed using Transmission Electron Microscopy (H-700, Hitachi Ltd., Tokyo, Japan).

#### 3.2.2. Microbiological Studies

##### Microdilution Assay

Following our previously published protocol [21,26,38,55,90,91], the microdilution standard method was conducted to assess MIC and MBC values for the IONP-CTAB colloidal dispersion against tested bacteria following Clinical and Laboratory Standards Institute guidelines [92]. The colloidal dispersion of NPs was serially diluted using sterile MHB in a 96-well microplate. A freshly prepared (10 µL) bacterium inoculum (6 log CFU/mL) of each tested bacterium was inoculated into each well, followed by overnight aerobic incubation of plates at 37 °C. Afterward, MTT solution (10 µL, 5 mg/mL, PBS) was inoculated into each well, and the plates were further incubated for 2 h at 37 °C, followed by addition of DMSO (100 µL/well) and incubation for another 2 h at room temperature to solubilize the formed formazan crystals. Subsequently, the absorbance of plates was measured at 570 nm using a Microplate Reader Spectrophotometer (Bio-Tek, MQX200, uQuant, Bio Tek instruments, Winooski, VT, USA). MIC was defined as the lowest concentration of nanoparticle colloidal dispersion required to inhibit bacterial growth, while MBC was identified as the lowest concentration of nanoparticle colloidal dispersion required to kill bacteria as visualized by absence of bacterial colonies when the inoculum taken from wells was plated onto Muller–Hinton agar (MHA) plates.

Several controls for each bacterium were prepared to assure the reliability of microbiological study: (1) negative control 1: sterile MHB culture media devoid of colloidal dispersion of NPs and bacteria; (2) negative control 2: sterile MHB culture medium containing serial dilutions of nanoparticles free of bacteria; and (3) positive control: MHB culture media inoculated with bacteria only. Afterward, the ratio of MBC to MIC was calculated for each tested bacterium to assess the bactericidal or bacteriostatic effect of IONPs-CTAB [48].

##### Time–Kill Curve Study of IONPs-CTAB

To assess the timeline of the antibacterial activity of nanoparticle colloidal dispersion, a time–kill curve study was conducted following our previously published protocol [7,26,42,55]. In brief, a fresh culture of tested bacteria (6 log CFU/mL in MHB) was individually subjected to nanoparticle colloidal dispersion at two concentrations: 125 µg/mL and 500 µg/mL. An aliquot of the inoculum was withdrawn at a predetermined time interval and serially diluted in a sterile diluent composed of 0.9% NaCl and 1% peptone. From each dilution, 100 µL was aseptically spread onto the surface of pre-dried MHA plates, followed by incubation for 18–24 h at 37 °C. After incubation, plates were examined for bacterial growth, and colonies were enumerated and compared to the control group (bacterial culture in absence of nanoparticle colloidal dispersion). Subsequently, growth inhibition was calculated relative to the control treatment.

##### Transmission Electron Microscopy (TEM) Study

A TEM study was conducted to investigate the ultrastructural changes that resulted from treating tested bacteria with IONPs-CTAB. According to our previously published methodology [7,26,42,55], a previously prepared overnight fresh culture (6 log CFU/mL in MHB) of tested bacteria was individually incubated with the nanoparticle colloidal dispersion (125 µg/mL) for 18–24 h. Subsequently, the bacterial suspension was centrifuged, and the resulting pellets were collected and washed twice with PBS (1.5 mL, pH 7.2) and then fixed in glutaraldehyde in PBS (2% *v*/*v*). Subsequently, post-fixation was carried out for 1 h at room temperature using osmium tetroxide (OsO_4_, 1% *w*/*v*) in PBS (5 mmol/L), and samples were rinsed thoroughly in PBS and dehydrated through a graded ethanol series before being embedded in epoxy resin. Semi-thin sections (500–1000 µm) were produced using a Leica ultra-microtome and stained with toluidine blue for a preliminary light microscopy examination using a 1× magnification lens (Leica (Wetzlar, Germany) ICC50 HD camera). Subsequently, ultra-thin sections (75–90 µm) were prepared and stained sequentially with saturated uranyl acetate and lead citrate. These sections were examined and imaged using JEM-1400 TEM (JEOL-Hitachi, Tokyo, Japan) at various magnifications. Image acquisitions were conducted with an AMT CCD Optronics camera (1632 × 1632-pixel format) equipped with a 1394 FireWire interface.

#### 3.2.3. In Vivo Study

##### Animals

For acute irritation study, New Zealand white rabbits (2.5 to 3 kg) were used to track the incidence of irritation in eyes and skin (intact/abraded) following a topical application of nanoparticle colloidal dispersion (250 µg/mL). For the wound healing study, adult male Sprague Dawley rats (180 and 200 gm) were used. All animals were obtained from CLAVCAP-VACSER, Cairo, Egypt.

Animals were housed under controlled environmental conditions including a 12 h light/dark cycle, a temperature of 25 ± 2 °C, and a relative humidity of 50 ± 20%. A standard commercial pelleted diet containing at least 5% fiber, 20% protein, 3.5% fat, 6.5% vitamins, ash and water was provided ad libitum. A one-week acclimatization period under strict hygienic conditions was allowed prior to initiating the study. All experimental procedures were conducted in accordance with approved ethical guidelines. For skin and eye irritation studies, protocols were reviewed and approved by the Ethics Committee of the Faculty of Veterinary Medicine at Cairo University (Vet CU20092022459). For the wound healing study, the protocol was reviewed and authorized by the Ethics Committee of the Faculty of Pharmacy, Capital University, Cairo, Egypt (IACUC: Approval No 6A2026). All efforts were conducted to ensure minimum animal discomfort throughout the study.

##### Acute Irritation Study

Skin Irritation Test

The skin irritation test was conducted following our previously established and published methodology [7,38,55,93]. A 1.5 × 1.5 cm^2^ section of the animals’ dorsal skin was marked and shaved with an electric razor 24 h prior to treatment. A total of 24 rabbits were randomly assigned into two main groups: (1) an intact skin group, in which the dorsal skin remained normal without injury, and (2) an abraded skin group, in which the stratum corneum was superficially disrupted using sterile needle scratches without causing bleeding, following standard skin irritation testing procedures. Each main group (12 rabbits) was further divided into three subgroups, each with four rabbits. Subgroup I: the untreated negative control; Subgroup II: rabbits that received 0.5 mL of blank sample (PBS, 10 mM, pH 7.4), vehicle used to disperse nanoparticles; Subgroup III: rabbits treated with 0.5 mL of nanoparticle colloidal dispersion at a concentration of 250 µg/mL. All substances were added topically on the skin using scaled dropper. During application, the animal was gently positioned in lateral recumbency to ensure accurate topical administration and to prevent leakage or loss of the applied material. This procedure was repeated three times for each application session to ensure uniform distribution and consistency of exposure. Following application, the designated skin sites were covered with sterile gauze of the same size and secured using non-irritant adhesive tape. Animals were investigated for erythema and edema at 24, 48, and 72 h using the Magnusson and Kligman scale (Magnusson and Kligman 1970 [94]). At 72 h post-treatment, all animals were photographed, euthanized by decapitation, and the skin treated was excised for histological analysis.

Eye Irritation Test

The modified Draize test was carried out following our previously established procedure [38,55,93] to assess the potential of ocular irritation induced by the application of nanoparticle colloidal dispersion (250 µg/mL) in comparison to the blank sample (PBS, 10 mM, pH 7.4). A total of 10 white New Zealand rabbits were used, with five animals assigned to assess each test material [95]. For administration, 50 µL of the designated sample was instilled into the lower conjunctival cul-de-sac of the left eye. Each eye received five instillations at 5 min intervals, while the right eye remained untreated and served as a negative control. The animals’ eyes were delicately closed for approximately 10 s to avoid sample loss. Following instillation, the eye lids were gently closed for about 10 s to minimize sample loss. Ocular responses were assessed at 24 and 72 h post-exposure using a histological grading system [96]. Clinical signs including erythema, conjunctival chemosis, discharge, and lesions of the cornea and iris were scored on a 0 to 3 scale where higher values indicate more severe irritation [97]. At 72 h post-treatment, all animals were photographed then euthanized by decapitation, and the ocular tissues were collected for histopathological examination.

Wound Healing Study

Excision Wound Model and Induction of Wound Infection

Rats were anesthetized with ketamine (100 mg/kg, i.p.) [98], and their dorsal hair was cleaned with 70% ethanol and shaved with an electric shaver. Using a scalpel and sharp scissors, a single full-thickness excisional circular wound with a diameter of 8 mm was made on each rat’s upper back under sterile conditions. The rats were then divided into two main groups: the infected group and the non-infected group. For the infected group, a bacterial broth containing 10 log CFU/mL of *S. aureus* was applied to the wound to induce infection. The bacterial broth was applied to the wounds five times a day until pus appeared, signifying the existence of infected wounds following previously published protocols [7,99,100].

Design of Experiments

Forty-eight injured rats were randomly assigned to eight groups; each group contained six injured animals, where each rat was housed individually in a cage. Afterward, four groups were assigned to assess the wound healing activity of IONPs-CTAB on non-infected wounds, while the remaining four groups were used to assess the wound healing activity of IONPs-CTAB on infected wounds. *S. aureus* was applied to induce wound infection as previously described. Each set group, non-infected versus infected, included the following treatment categories: (1) untreated group, (2) blank group, (3) IONP-CTAB group, and (4) standard treatment group. For a non-infected wound, the standard treatment was Mebo^®^ ointment [78], whereas Garamycin^®^ cream was used as a standard treatment for the infected wound [101]. *S. aureus* used in the current study was sensitive to Gentamicin, as confirmed by the antibiotic susceptibility test (Table S1 in the Supplementary Materials). IONPs-CTAB, blank, Mebo^®^ ointment, or Garamycin^®^ cream were applied topically to the wound site once daily, as summarized in Table 4.

The wound area was photographed and recorded at predetermined time intervals. Three distinct axes were used to measure the wound diameter to assess the progression of wound closure and, consequently, wound healing. Wound closure percentage was calculated using the following equation to provide a quantitative assessment of the wound healing efficacy of each treatment.

Wound closure percentage = [(Initial wound diameter − wound diameter measured at specified time interval)/Initial wound diameter] × 100.

Animals were sacrificed on day 14 and 18 for non-infected and infected wounds, respectively. Skin samples were excised for histopathological examination and biochemical analysis, including wound healing markers. The skin tissue taken from the wound site was cleaned of blood and separated into two sections. One section was frozen at −80 °C for biochemical analysis, while the other was preserved in 10% neutral buffer formalin for histopathological examination.

Histopathological Examination

Tissues of intact/abraded skin, eyes, and wound sites were collected, rinsed with ice-cold PBS, fixed in 10% neutral buffered formalin, processed routinely, and stained with hematoxylin and eosin (H&E) following the previously published protocol [102].

For the acute irritation study, tissue histological alterations were assessed using a validated semi-quantitative scoring system (0–3, 0 = no anomaly, 1 = little abnormality, 2 = mild abnormality, and 3 = substantial abnormality), covering seven parameters: hypertrophy, hyperkeratosis, parakeratosis, erosion, inflammatory cell infiltration, extracellular edema, and ulceration [102].

For wound healing assessment, collagen deposition was quantified using Masson’s trichrome staining [7,102,103,104]. A light microscope with an installed digital camera was used for examination and capturing micrographs (Olympus, Tokyo, Japan, Cell Sens), where six non-overlapping microscopic fields of stained sections were randomly scanned. The area percentage of reactive collagen fibers was measured using Image Analysis Software (Image J, 1.46a, NIH, Bethesda, MD, USA). All evaluations were performed under blind conditions.

##### Biochemical Analysis

Skin Tissue Preparation

Skin tissues were rinsed with ice-cold saline, blotted dry, and weighed. A 10% (*w*/*v*) tissue homogenate was prepared in ice-cold phosphate-buffered saline (0.1 M, pH 7.4) and centrifuged at 3000 rpm for 30 min at 4 °C. The resulting supernatant was collected for subsequent chemical analyses.

Lipid Peroxidation Determination

Malondialdehyde (MDA), a marker of lipid peroxidation, was quantified in granulation tissue using a commercial assay kit (BioVision, Cat.No. K739-100, Milpitas, CA, USA) according to the manufacturer’s protocol.

Identification of Inflammatory Markers

Levels of interleukin-1β (IL-1β) and tumor necrosis factor-alpha (TNF-α) were quantified using commercial enzyme-linked immunosorbent assay (ELISA) kits following the manufacturer’s instructions. IL-1β was measured using a kit from CUSABIO Life Sciences (Cat. No. CSB-E11987r, Wuhan, China), while TNF-α was assessed using a kit from MyBioSource (Cat. No. MBS825017, San Diego, CA, USA).

Assessment of Growth Factors

Transforming growth factor-beta1 (TGF-β1) and vascular endothelial growth factor (VEGF) were quantified using commercial ELISA kits supplied by MyBiosource, Inc. (Cat. No. MBS824788 and MBS724516, San Diego, CA, USA) following the manufacturers’ instructions.

Assessment of Hydroxyproline Content

Hydroxyproline, an essential marker of collagen deposition and wound repair, was measured using an ELISA kit supplied by MyBioSource (Cat. No. MBS017427, San Diego, CA, USA) following the manufacturer’s instructions.

#### 3.2.4. Statistical Analysis

Statistical analysis and graphical outputs were generated using GraphPad Prism, version 9 (GraphPad Software Inc., San Diego, CA, USA). Comparisons among multiple groups were carried out using one-way analysis of variance (ANOVA), followed by Tukey’s test. For wound closure percent, two-way ANOVA followed by the Bonferroni test was applied. Differences were considered statistically significant at *p* < 0.05.

## 4. Conclusions

The current study assesses the potential topical application of chemically synthesized IONPs-CTAB to enhance wound healing of both non-infected and infected wounds. Based on the antibacterial activity studies, IONPs-CTAB had MIC values ranging from 125 to 250 µg/mL and MBC values ranging from 500 to 1000 µg/mL against the tested bacteria: *S. aureus*, MRSA, and *E coli*. The time–kill curve study demonstrated a more efficient antibacterial activity of IONPs-CTAB at 125 µg/mL compared to 500 µg/mL (4×MIC); this was linked to NP aggregation at a high concentration. Moreover, the acute irritation test revealed that IONPs-CTAB were incompatible with the abraded skin and eyes of rabbits after topical application at 250 µg/mL, contrary to their biocompatibility at 125 µg/mL with the skin and eyes of rabbits. The wound healing study conducted at 125 µg/mL revealed successful wound healing at day 14 and day 18 for both non-infected and infected wounds, respectively. This was further confirmed through histopathological examination showing the extra-deposition of collagen in the wounds treated with IONPs-CTAB compared to untreated animals and those treated with standard treatment. This was further confirmed through biochemical analysis, where IONPs-CTAB showed lower levels of reactive oxygen species markers (MDA content) and reduced levels of inflammatory mediators such as TNF-α and IL-1β compared to untreated animals and those treated with standard therapy. Moreover, IONPs-CTAB were found to enhance tissue regeneration and matrix remodeling by upregulating VEGF, TGF-β1, and hydroxyproline contents. Based on our findings, IONPs-CTAB are highly recommended for the therapeutic management of non-infected and infected wounds. However, future studies are still required to assess their long-term safety and the possibility of their extravasation to systemic circulation, with their potential accumulation in various organs after a long-term application.

## Figures and Tables

**Table 1:** MIC and MBC (µg/mL) values of IONPs-CTAB recorded against tested bacterial pathogens. MBC/MIC value was calculated to assess the bactericidal/bacteriostatic action of IONPs-CTAB.

Pathogen	MIC	MBC	MBC/MIC Value
*S. aureus*	125	500	4
MRSA	125	500	4
*E. coli*	250	1000	4

MIC, minimum inhibitory concentration; MBC, minimum bactericidal concentration.

**Table 2:** Evaluation of skin irritation following treatment with IONP-CTAB colloidal dispersion (250 µg/mL) and its blank.

		Control Site								Treated Site							
		Erythema and Eschar				Edema				Erythema and Eschar				Edema			
		Intact		Abraded		Intact		Abraded		Intact		Abraded		Intact		Abraded	
Tested sample/tested time (h)	Animal Number	24	72	24	72	24	72	24	72	24	72	24	72	24	72	24	72
Untreated	1	0	0	0	0	0	0	0	0	0	0	0	0	0	0	0	0
	2	0	0	0	0	0	0	0	0	0	0	0	0	0	0	0	0
	3	0	0	0	0	0	0	0	0	0	0	0	0	0	0	0	0
IONPs-CTAB (250 µg/mL)	4	0	0	0	0	0	0	0	0	0	0	1	2	0	0	0	1
	5	0	0	0	0	0	0	0	0	0	0	1	1	0	0	0	1
	6	0	0	0	0	0	0	0	0	0	0	0	1	0	0	0	1
Blank (PBS: 10 mM, pH 7.4)	7	0	0	0	0	0	0	0	0	0	0	0	0	0	0	0	0
	8	0	0	0	0	0	0	0	0	0	0	0	0	0	0	0	0
	9	0	0	0	0	0	0	0	0	0	0	0	0	0	0	0	0

All skin irritation ratings were 0 after 24 and 72 h, both intact and abraded skin showed no symptoms (erythema, eschar, and edema) indicating incidence of irritation for untreated and animals treated with blank (PBS: 10 mM, pH 7.4, vehicle used to disperse IONPs-CTAB). IONPs-CTAB (250 µg/mL) showed different scores in abraded site, indicating their irritating nature for the abraded skin at investigated concentration, 250 µg/mL.

**Table 3:** Eye irritations were assessed following treatment with IONP-CTAB colloidal dispersion (250 µg/mL) compared to blank and untreated eyes.

Tested Solution	Tissues Examined in the Eye	Number of Rabbits					
		1		2		3	
		RT. Untreated	LT. Treated	RT. Untreated	LT. Treated	RT. Untreated	LT. Treated
Untreated	Cornea	0	0	0	0	0	0
	Iris	0	0	0	0	0	0
	Conjunctiva	0	0	0	0	0	0
IONPs-CTAB (250 µg/mL)	Cornea	0	2	0	2	0	2
	Iris	0	0	0	0	0	0
	Conjunctiva	0	2	0	1	0	1
Blank (PBS: 10 mM, pH 7.4)	Cornea	0	0	0	0	0	0
	Iris	0	0	0	0	0	0
	Conjunctiva	0	0	0	0	0	0

**Table 4:** Treatment options for non-infected and infected wounds.

Subgroups for Non-Infected and *S. aureus*-Infected Wounds	Treatment Options
Untreated group	Rats’ wounds were left untreated; instead, they were allowed to mend naturally without the use of any medication.
Blank group	Once a day, 40 µL of the vehicle (PBS, 10 mM, pH 7.4) used to disperse IONPs-CTAB was applied to the wound.
IONPs-CTAB group	Once a day, IONPs-CTAB (40 µL, 125 µg/mL) were applied to the wound, and the amount of IONPs-CTAB per square millimeter of wound was 0.1 µg/mm^2^.
Mebo^®^ ointment (standard treatment for a non-infected wound)	Once a day, 0.5 g of the commercially available wound-healing agent Mebo^®^ ointment was applied to the wound.
Garamycin^®^ cream (standard treatment for an infected wound)	Once a day, 0.5 g of the commercially available Garamycin^®^ cream was applied to the infected wound.

## Data Availability

All authors are happy to share all data (including supplementary data). Further inquiries can be directed to the corresponding author.

## Supplementary Materials

The following supporting information can be downloaded at: https://www.mdpi.com/article/10.3390/antibiotics15060584/s1, Figure S1: FTIR spectra for IONPs without CTAB, and IONPs-CTAB, and CTAB; Figure S2: DSC-TGA thermographs for (A) IONPs coated with CTAB and (B) IONPs without CTAB; Figure S3: XPS analysis for IONPs without CTAB and IONPS coated with CTAB; Table S1: Antibiotic susceptibility profile (ASP) of tested bacteria according to CLSI (2016).
