# Supplementary material for: Wound Healing Activity of Iron Oxide Nanoparticles; Comparative In Vivo Study on Staphylococcus aureus-Infected and Non-Infected Wounds
Source: Antibiotics (Basel). 2026 Jun 8;15(6):584. doi: 10.3390/antibiotics15060584 (PMC13295391; doi:10.3390/antibiotics15060584)
Supplement: Supplementary file 1 [file antibiotics-15-00584-s001.zip › antibiotics-4277930-supplementary.pdf]

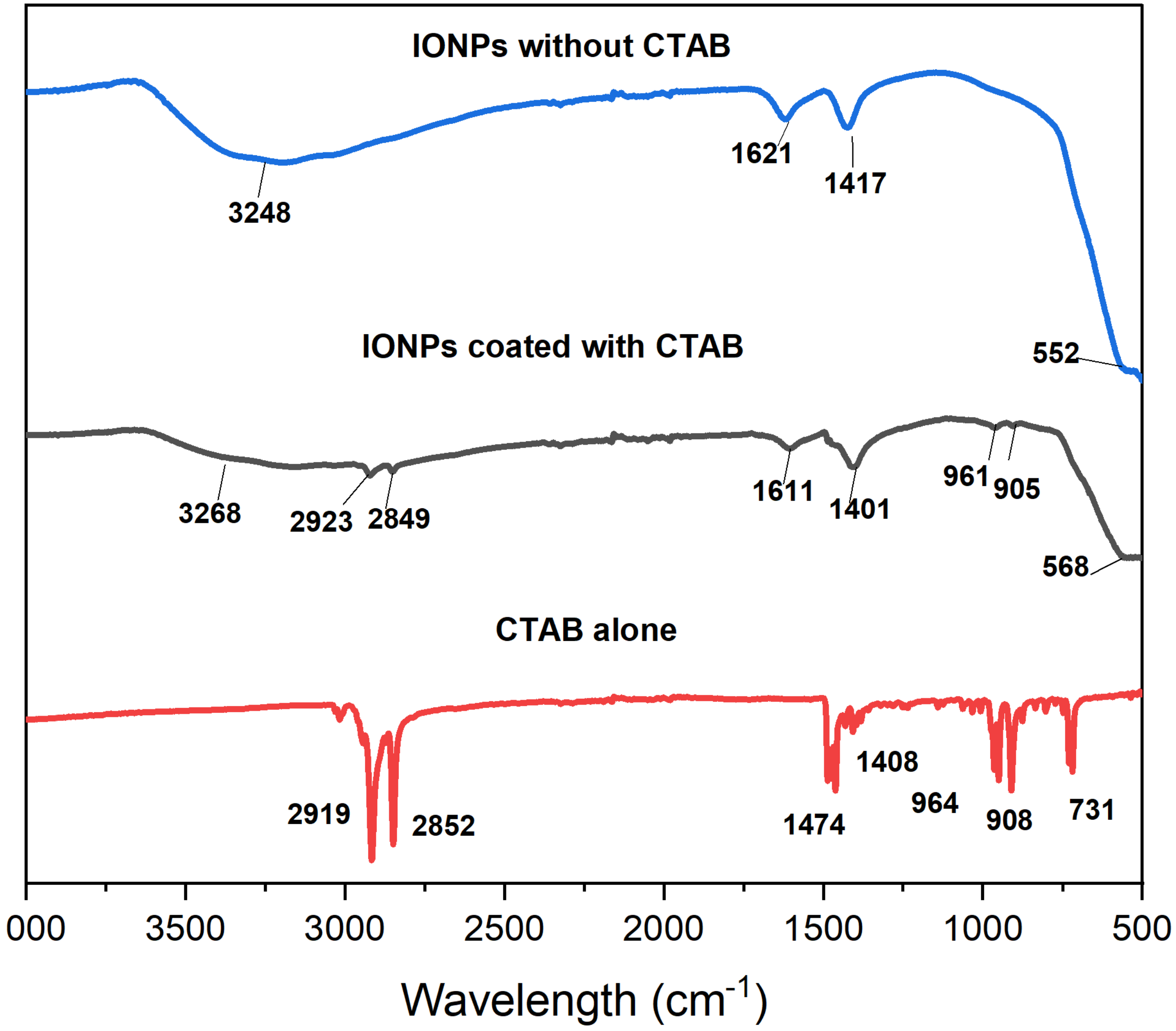

Figure S1: FTIR spectra for IONPs without CTAB, and IONPs-CTAB, and CTAB.

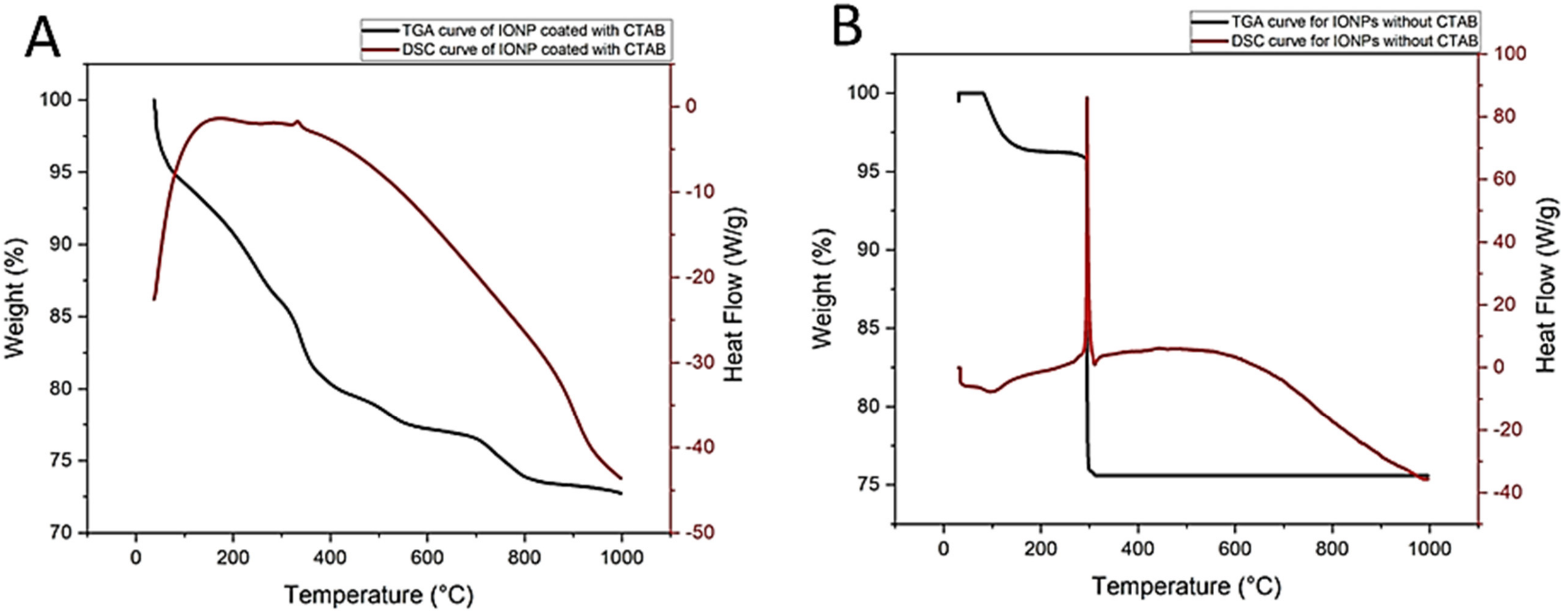

Figure S2: DSC-TGA thermographs for (A) IONPs coated with CTAB and (B) IONPs without CTAB

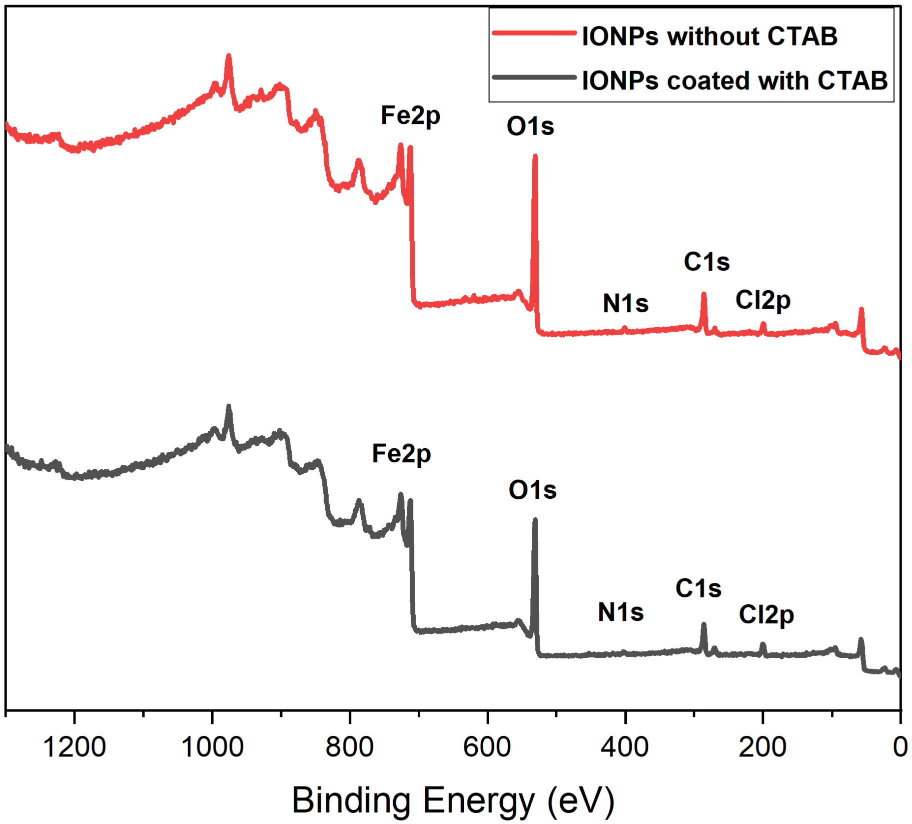

Figure S3: XPS analysis for IONPs without CTAB and IONPS coated with CTAB

Table S1: Antibiotic susceptibility profile (ASP) of tested bacteria according to CLSI (2016).

Bacterial strains	Chemical group of Antibiotics (µg)																
	Aminoglycoside		ß-lactams			Cephems								Glycopeptides	Quinolones		Sulfonamide
	GMN$_{10}$	KAN$_{30}$	AMP$_{10}$	P$_{10}$	OX$_{30}$	FOX$_{30}$	CRO$_{30}$	CFM$_{5}$	CEC$_{30}$	CFP$_{75}$	CFR$_{30}$	CXM$_{30}$	CFS$_{75/30}$	VA$_{30}$	NOR$_{10}$	OFX$_{5}$	SXT$_{25}$
S. aureus	S	S	R	R	S	R	R	R	R	R	R	R	R	S	R	S	S
MRSA	R	R	R	R	R	R	R	R	R	R	R	R	R	R	R	S	S
E. coli	S	R	R	R	R	R	R	R	R	R	R	R	R	nt	R	R	R

R; resistant, S; sensitive, nt; not tested.

List of antibiotic name abbreviations

GMN: Gentamycin	OX: Oxacillin	CEC: Cefaclor	CFS: Cefoperazone/sublactam
KAN: Kanamycin	FOX: Cefoxitin	CFP: Cefoperazone	VA: Vancomycin
AMP: Ampicillin	CRO: Ceftriaxone	CFR: Cephalosporine	NOR: Norfloxacin
P: Penicillin	CFM: Cefixime	CXM: Cefuroxime	OFX: Oflatoxin
			SXT: Trimethoprim/Sulfamethoxazole
